# Electrochemical oxygen reduction to hydrogen peroxide at practical rates in strong acidic media

**DOI:** 10.1038/s41467-022-30337-0

**Published:** 2022-05-24

**Authors:** Xiao Zhang, Xunhua Zhao, Peng Zhu, Zachary Adler, Zhen-Yu Wu, Yuanyue Liu, Haotian Wang

**Affiliations:** 1 Department of Chemical and Biomolecular Engineering, Rice University, Houston, TX 77005 USA; 2 Texas Materials Institute and Department of Mechanical Engineering, The University of Texas at Austin, Austin, TX 78712 USA; 3 Department of Chemistry, Rice University, Houston, TX 77005 USA; 4 Department of Materials Science and NanoEngineering, Rice University, Houston, TX 77005 USA

**Keywords:** Electrocatalysis, Chemical engineering, Electrocatalysis, Catalytic mechanisms

## Abstract

Electrochemical oxygen reduction to hydrogen peroxide (H_2_O_2_) in acidic media, especially in proton exchange membrane (PEM) electrode assembly reactors, suffers from low selectivity and the lack of low-cost catalysts. Here we present a cation-regulated interfacial engineering approach to promote the H_2_O_2_ selectivity (over 80%) under industrial-relevant generation rates (over 400 mA cm^−2^) in strong acidic media using just carbon black catalyst and a small number of alkali metal cations, representing a 25-fold improvement compared to that without cation additives. Our density functional theory simulation suggests a “shielding effect” of alkali metal cations which squeeze away the catalyst/electrolyte interfacial protons and thus prevent further reduction of generated H_2_O_2_ to water. A double-PEM solid electrolyte reactor was further developed to realize a continuous, selective (∼90%) and stable (over 500 hours) generation of H_2_O_2_ via implementing this cation effect for practical applications.

## Abstract (web summary)

Electrochemical oxygen reduction to H_2_O_2_ in acidic media suffers from low selectivity, especially at high current densities. Here, the authors report a cation-regulated “shielding effect” to promote the H_2_O_2_ selectivity under industrial-relevant current in strong acid.

## Introduction

Hydrogen peroxide (H_2_O_2_) is ranked as one of the top 10 most energy-intensive chemicals in the chemical manufacturing bandwidth study by the Advanced Manufacturing Office in Department of Energy^1^. It is currently manufactured industrially by the energy- and waste-intensive anthraquinone cycling process^2–4^, which consumes a primary current typical energy of ~13,000 Btu/lb (8.1 kWh/kg) without taking into account the H_2_/O_2_ feedstocks^1^. Electrochemical synthesis of H_2_O_2_ via the 2e^−^ oxygen reduction reaction (2e^−^–ORR), where the O_2_ molecule is electrochemically reduced to H_2_O_2_ via a two-electron (2e^−^) pathway, provides a promising energy-efficient and low-waste alternative^2–7^. Recent efforts have been mostly focused on developing catalysts in alkaline solutions, in which small overpotential and high selectivity of 2e^−^–ORR toward H_2_O_2_ have been comparatively easy to achieve on low-cost materials such as carbon^8–18^. However, in alkaline solutions, H_2_O_2_ is deprotonated (p*K*_*a*_ > 11) and easily degraded^19^. Moreover, for practical electrolyzers such as membrane electrode assembly (MEA), catalysts developed in alkaline solutions need to be applied on an anion exchange membrane (AEM), which is typically not as stable as its counterpart of proton exchange membrane (PEM), e.g., Nafion^4^, especially operated under air. In addition, with stronger oxidation ability in acid, the acidic H_2_O_2_ solution shows a wider range of applications and greater demand^6,20^, which strongly motivates studies in high-performance electrochemical generation of H_2_O_2_ in acidic media.

**Fig. 1:**
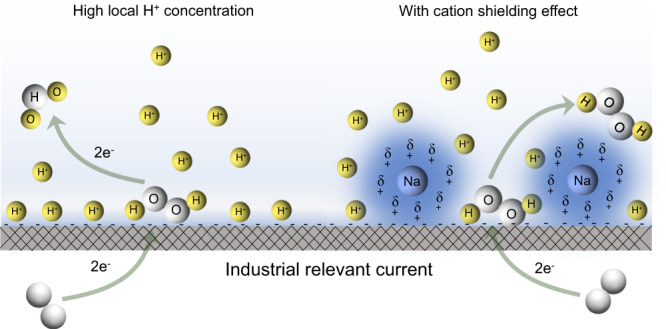
Schematic illustration of 2e^−^–ORR toward H_2_O_2_ in acid with/without Na^+^ under industrial-relevant current. Here we report a cation-regulated catalyst/electrolyte interface to promote electrochemical O_2_ reduction to H_2_O_2_ in acids with high-selectivity and industrial-relevant production rates. By adding only a small amount of alkali metal ions into the acidic electrolyte, which barely affects the solution’s pH, we demonstrated a dramatic improvement in H_2_O_2_ selectivity and activity especially under large ORR current densities across different catalysts. Our molecular dynamic simulations suggest that the solvated alkali metal cations, compared to concentrated protons in acids, could preferentially be attracted to the catalyst/electrolyte interface and squeeze out local protons during the reaction, suppressing the further reduction of as-generated H_2_O_2_ to H_2_O (Fig. 1). Using commercial carbon black catalysts with 10 mM Na_2_SO_4_ as an additive, the H_2_O_2_ Faradaic efficiency (FE) can reach over 80% under a significant current of 400 mA cm^−2^ in 0.1 M H_2_SO_4_, representing a 25-fold improvement compared to the case without Na^+^ additive where negligible H_2_O_2_ was produced (<5% FE). Based on this cation promotion concept, a double-PEM-based solid electrolyte (SE) reactor was developed for a continuous generation of H_2_O_2_ with high FE (∼90%) and good stability (over 500 h) for practical applications in the future.

Till so far, there are only a few known noble metal catalysts, including Pt- and Pd-based catalysts, demonstrated to be selective and stable for 2e^−^–ORR in strong acids^21–24^, but their high cost and toxicity of heavy metals (in the case of PtHg alloy^22,23^) could limit their applications in large-scale H_2_O_2_ generation. Some low-cost catalysts such as carbon materials may also show good H_2_O_2_ selectivity in acids within small overpotential and small current density regions (typically less than 10 mA cm^–^^2^)^25–29^, but their H_2_O_2_ selectivity and stability were dramatically dropped when an industrial-relevant current was reached^5,27,30^. In acidic media, carbon catalysts present sluggish ORR kinetics and typically require a large overpotential (>300 mV) to initiate the ORR reaction, and consequently, a large negative cathodic overpotential is required to deliver a high current density^26–29^. While the carbon surface might intrinsically prefer a 2e^−^–ORR pathway due to their relatively weak binding with oxygen intermediates as demonstrated by several previous studies^26,27,30,31^, such negative overpotential could further push the ORR reaction all the way down to H_2_O with significantly decreased H_2_O_2_ selectivity and production rate especially in acids. This is because under negative potentials in acids, the catalyst surface accumulates concentrated protons that are prone to further reduce the locally generated H_2_O_2_ molecules to H_2_O (H_2_O_2_ + 2e^−^ + 2H^+^ = 2H_2_O). Therefore, diluting the local proton concentration and minimizing the electrochemical dissociation of as-produced H_2_O_2_ to H_2_O could be one promising strategy for resolving this H_2_O_2_ selectivity-activity dilemma, and delivering industrial-relevant H_2_O_2_ production rate in acidic solution while maintaining good H_2_O_2_ selectivity^32,33^ (Fig. 1).

## Results

### The alkali metal cation effect on acidic H_2_O_2_ generation in flow cell

**Fig. 2:**
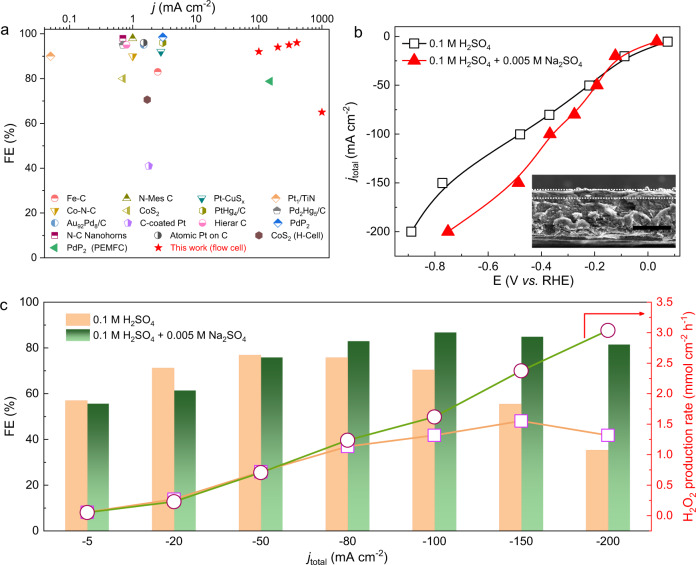
Na^+^ effects toward acidic H_2_O_2_ generation through 2e^−^–ORR. **a** Reported current densities and Faradaic efficiencies (FEs) for 2e^−^–ORR toward H_2_O_2_ in acid in literatures (listed in Table S1). **b** Effect of 0.005 M Na_2_SO_4_ on the total activity over carbon catalysts BP2000 in a flow cell. The cell voltages were 85% iR compensated. The inset is the cross-section SEM image of the electrode. The scale bar is 200 µm. **c** The comparison of H_2_O_2_ FE and production rate over carbon catalyst BP2000 with/without 0.005 M Na_2_SO_4_ in a flow cell. The Na^+^ serves as a promoter for the production of H_2_O_2_ through 2e^−^–ORR. The H_2_O_2_ FE and production rate are both increased, especially at the high current range.

**Fig. 3:**
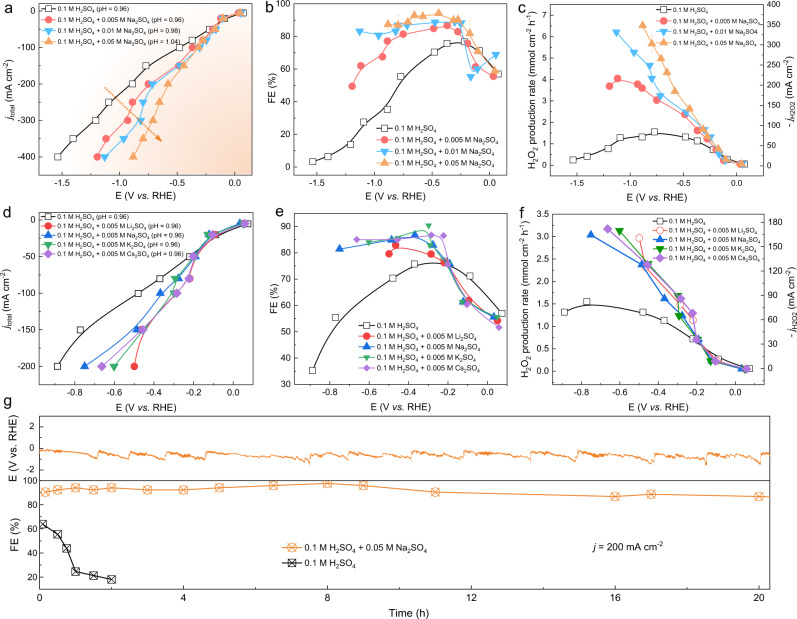
The effect of alkali metal cation concentration and species on electrosynthesis of H_2_O_2_ through 2e^−^–ORR. **a** The I-V curve of ORR with different concentrations of cations (from 0 to 0.05 M Na_2_SO_4_) in a flow cell. **b** The corresponding FEs and (**c**) production rates and partial current of H_2_O_2_ products under different cell voltages. With the increase of cation concentration, the H_2_O_2_ FE and production rate continuously increase at high current densities. The error bars represent two independent tests. **d** The I-V curve of ORR in 0.1 M H_2_SO_4_ containing different types of cations (0.005 M X_2_SO_4_, X = Li^+^, Na^+^, K^+^, Cs^+^) in a flow electrolyte cell. **e** The corresponding FEs and (**f**) production rates and partial current of H_2_O_2_ products under different cell voltages. **g** Chronopotentiometry stability comparison of carbon catalysts BP2000 with/without cations by holding 200 mA·cm^−2^ current density for 20 h in a flow cell for continuous electrolysis. The geometric area of the flow field at the cathode in the flow cell is 1 cm^2^.

We first employed a standard three-electrode flow cell reactor to investigate the cation effect towards H_2_O_2_ generation in acid (Fig. S1), allowing to evaluate our hypothesis in a more practical environment and produce H_2_O_2_ under higher current densities compared to the traditional RRDE setup (Supplementary Note 1, Fig. 2a). The commercially available carbon black catalyst (BP2000) with a high surface area was used as a model ORR catalyst in this study (Fig. S2). Its intrinsic H_2_O_2_ activity and selectivity were first evaluated in 0.1 M H_2_SO_4_ electrolyte (pH = 0.96) (Fig. 2b, c). We observed that while the H_2_O_2_ FE of carbon black catalyst in acid remained relatively good (~70%) under small current regions, it started to decrease dramatically once the current density is over 100 mA cm^−2^ with very negative applied potentials (Fig. 2c). Under 200 mA cm^−2^, the carbon black catalyst can only deliver a 35% H_2_O_2_ FE and the majority of electrons were directed towards H_2_O instead (Fig. 2c). The decreased FE is as expected, because under such a negative potential of −0.89 V versus reversible hydrogen electrode (vs. RHE) needed to drive this high current, even if the catalyst prefers to reduce O_2_ to H_2_O_2_ in its first place, those generated H_2_O_2_ at the electrode surface could be easily further reduced to H_2_O coupling two electrons and two local protons. Please be noted here that, under each current density, its corresponding H_2_O_2_ FE was measured within 8 min of operation. With a longer time of electrolysis, the H_2_O_2_ FE could be further dropped (as shown in the stability test in Fig. 3g). After introducing a trace amount of Na^+^ additive (5 mM Na_2_SO_4_) into the acidic electrolyte, while the ORR activity did not show that much difference (Fig. 2b), the H_2_O_2_ FE was significantly improved especially under high current densities. As shown in Fig. 2c, in the potential range of the ORR onset, the impact of Na^+^ (5 mM Na_2_SO_4_) toward H_2_O_2_ is negligible; while in the range of large overpotentials at high current densities, the small amount of Na^+^ helped the carbon black catalyst to hold a high H_2_O_2_ FE plateau of over 80% until 200 mA cm^−2^, suggesting a more than doubled FE compared to that in pure acid (Fig. 2c).

The measured trends of H_2_O_2_ FE show that the promotion effect of Na^+^ is more pronounced at high current density toward H_2_O_2_ formation. We understand that Na^+^ and protons are both positively charged ions and will be attracted towards the ORR catalyst surface to form the electrochemical double layer under negative potentials^34^. We would assume that, Na^+^ ions could be more competitive than protons to be aligned along the electrochemical double layer, which dramatically reduces the local proton concentration and thus protects the generated H_2_O_2_ from further reductions coupling protons and electrons (discussed in the following sections in detail). To further amplify the cation effect and drive the O_2_-to-H_2_O_2_ production at even higher current densities in acid (>200 mA cm^−2^), we gradually increased the Na^+^ cation concentration in the electrolyte. As shown in Fig. 3a, the overall current density, as well as the H_2_O_2_ FE gradually increases with increased Na^+^ concentrations. In general, a higher concentration of Na^+^ can maintain larger 2e^−^–ORR currents without sacrificing the H_2_O_2_ selectivity. With only 0.01 M Na_2_SO_4_ in 0.1 M H_2_SO_4_, the FE of H_2_O_2_ can reach 83% at 400 mA cm^−2^ (Fig. 3b), representing a 25-fold improvement compared to that in pure acids without Na^+^ (H_2_O_2_ FE only 3.3%). Along with the increased H_2_O_2_ FE, the improvement of the H_2_O_2_ production rate was also obvious at high current densities (Fig. 3c). For example, the production rate of 6.21 mmol cm^−2^ h^−1^ H_2_O_2_ (partial current of 332 mA cm^−2^) was achieved under the current density of 400 mA cm^−2^, much higher than that in pure H_2_SO_4_ acid (only 0.245 mmol cm^−2^ h^−1^). The production rate can be further enhanced by providing more Na^+^ cations (the production rate of 6.52 mmol cm^−2^ h^−1^ was achieved at 400 mA cm^−2^ and the maximum FE can be up to 94% at 150 mA cm^−2^ by using 0.05 M Na_2_SO_4_ as additive). Further increasing the concentration of Na^+^ cations could continually push up the H_2_O_2_ FE to higher values at high current densities (Fig. S3). It is important to note here that the electrolyte pH did not show obvious change and stayed around pH 1 after these Na_2_SO_4_ additives, ranging from 0.96 (0.1 M H_2_SO_4_), 0.96 (0.1 M H_2_SO_4_ + 0.005 M Na_2_SO_4_), 0.98 (0.1 M H_2_SO_4_ + 0.01 M Na_2_SO_4_) to 1.04 (0.1 M H_2_SO_4_ + 0.05 M Na_2_SO_4_). To fully exclude the pH effect of the bulk solution (even though the change is quite small), the pH value of 0.1 M H_2_SO_4_ + 0.1 M Na_2_SO_4_ (pH = 1.13) was adjusted to be the same as that of 0.1 M H_2_SO_4_ (pH = 0.96) by adding more sulfuric acid. As shown in Fig. S4 and S5, the H_2_O_2_ FE in both electrolytes with Na^+^ additives (before and after pH tuning) showed very similar trend in all current ranges, indicating the Na^+^ cations dominate the H_2_O_2_ generation process and the small pH differences of electrolytes have negligible influence on the production rate or H_2_O_2_ FE. With 0.1 M Na_2_SO_4_ as an additive in 0.2 M H_2_SO_4_ solution (pH = 0.76), we were able to produce H_2_O_2_ at the current density of 1 A cm^−2^ with a FE of more than 65% (Fig. S6). The H_2_O_2_ partial currents of up to 650 mA cm^−2^ were achieved, and high FEs were maintained, better than the highest O_2_-to-H_2_O_2_ conversion rates reported. At even lower pH electrolyte, i.e., 1 M H_2_SO_4_ solution (PH~0), similar trends were also observed (Fig. S7), indicating the general phenomenon of cation promotion effect toward H_2_O_2_ production through ORR.

The low threshold of the alkali metal cation concentration towards promoting H_2_O_2_ generation puts forward new requirements for the purity of the electrolyte during ORR to H_2_O_2_ tests in acids. For the traditional electrolytic ORR process, the Na_2_SO_4_ is widely used as the anolyte to balance the electrochemical reaction. However, even far away from the cathode side and separated by ion exchange membranes, we found that the Na^+^ in the anolyte can still penetrate the PEM and move to the cathode chamber, and thus significantly improve the H_2_O_2_ FE of ORR at the cathode (Fig. S8). Therefore, it is highly recommended to perform the ORR reaction using the same acidic electrolyte to avoid any cross-over cation contaminations which could significantly improve H_2_O_2_ performance in acids.

The promotion effect is not only limited to the Na^+^. The H_2_O_2_ production rate can also be enhanced by using other alkali metal ions. Figure 3d shows the I-V curves for four different alkali metal cations in each sulfuric acid electrolyte with a concentration of 0.01 M (0.005 M X_2_SO_4_, X = Li, Na, K, Cs). As compared with the pure H_2_SO_4_ electrolyte, while the ORR activities were slightly improved, significant improvements in H_2_O_2_ FE were observed for all the cations (Fig. 3d–f). The FE and production rates of H_2_O_2_ are relatively unaffected by the size of the alkali metal cations in the electrolyte. As differences in total current density exist for electrolytes containing different cations, the H_2_O_2_ production rate provides a better representation of trends in product formation rates than FEs. As shown in Fig. 3f, with 0.005 M X_2_SO_4_ as the additive, all the alkali metal cations are able to drive the O_2_-to-H_2_O_2_ reaction efficiently with high production rates, and the differences induced by different cations are relatively marginal. Nevertheless, we find that the promotion effect is only limited to IA alkali metal cations (such as Li^+^, Na^+^, K^+^, Cs^+^), while the other cations (including the IIA cations such as Mg^2+^, Ca^2+^ and IIIA cations such as Al^3+^) decrease the H_2_O_2_ FE dramatically. This might due to the local environment change from acid to alkaline induced by the cation additives during ORR (will discuss the details in the simulation part). The alkaline local environment could induce the formation of solid metal hydroxide on the catalyst surface and block the ORR reaction, decreasing the H_2_O_2_ FE and production rate (Fig. S9).

**Fig. 4:**
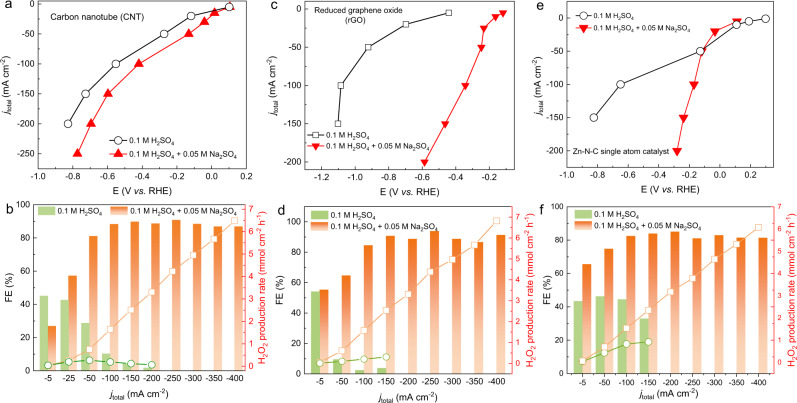
The Na^+^ effect towards electrochemical production of H_2_O_2_ on diverse carbon-based electrocatalysts. The I-V curve of ORR in 0.1 M H_2_SO_4_ or 0.1 H_2_SO_4_ + 0.05 M Na_2_SO_4_ electrolytes using (**a**) CNT, **c** rGO, **e** Zn-N-C single-atom catalysts. The corresponding FEs and production rates of H_2_O_2_ in 0.1 M H_2_SO_4_ or 0.1 H_2_SO_4_ + 0.05 M Na_2_SO_4_ electrolytes using (**b**) CNT, **d** rGO, **f** Zn-N-C single-atom catalysts.

It was also exciting to find out that the cation additives not only promote the H_2_O_2_ selectivity but also improve the long-term operation stability, which is another important target for practical production of H_2_O_2_. Figure 3g shows the comparison of H_2_O_2_ FE at 200 mA/cm^2^ ORR current as a function of operation time in the flow cell. In pure acidic electrolyte, the H_2_O_2_ FE rapidly dropped to less than 10% within 2 h. As a sharp contrast, with the presence of Na^+^ cations, the potential and H_2_O_2_ FE showed negligible changes for over 20 h. We suppose the improved activity and stability are induced by the alkalization of the local environment induced by the cation additives during ORR. During the ORR process, the solvated alkali metal cations, compared to concentrated protons in acids, could preferentially be attracted to the catalyst/electrolyte interface, which gives rise to a local alkaline environment (will discuss the details in the simulation part). At alkaline conditions, the carbon-based catalyst typically shows higher activity and better stability compared to acidic conditions^8–10^. We then found this cation promotion effect has broad applicability to different catalysts and acidic electrolytes. With a small amount of Na_2_SO_4_ as the additive in 0.1 M H_2_SO_4_ (Fig. 4), high H_2_O_2_ FE under high production rates can be achieved on various carbon catalysts including carbon nanotube (CNT), reduced graphene oxide (rGO), XC-72, and the carbon-based single-atom catalysts (e.g., Zn-N-C). This promotion effect is independent of the catalyst structures, surface morphology or surface area (Figs. S10–S12). In addition, this cation impact on 2e^−^–ORR also applies to other acidic electrolytes such as HClO_4_, and the Na^+^ sources can also come from other salts such as NaHSO_4_ (Figs. S13, S14).

### Mechanistic understanding of the cation effect on H_2_O_2_ selectivity

To further explore our aforementioned hypothesis and understand the cation effects on 2e^−^–ORR selectivity, we carried out constant-potential *ab*-initio molecular dynamics (AIMD) simulations^35^ based on DFT. In the simulations, we use a typical potential V_RHE_ (potential relative to RHE) = −1 V at which the cation effect is most prominent. The hydrogen evolution reaction was not considered because only a trace amount of H_2_ byproduct (from H_2_ evolution at large overpotentials) was detected from the cathode side (Fig. S15). We consider the acid condition and use pH = 0 in our simulations. Since alkali metal cations are not likely to directly participate in ORR, we explore two aspects of the cation effects: (1) how alkali metal cations affect the distribution of the protons, (2) how the redistribution of protons will influence the selectivity of O_2_ reduction to H_2_O_2_. For the first question, we adopted a model with relatively thick water layers (equivalent to 6 ice layers) on (6 × 6) periodic graphene (Fig. S16), and then put a certain number of cations/protons to represent the electrode environment under the lower potential like V_RHE_ = −1 V. For the second question, we use a thinner model (~4 ice layers) with single-vacancy to represent the reaction site, and then use slow-growth method^36^ to evaluate the reaction barrier under different conditions). The details of the simulations can be found in the experiment section in supporting information (SI).

**Fig. 5:**
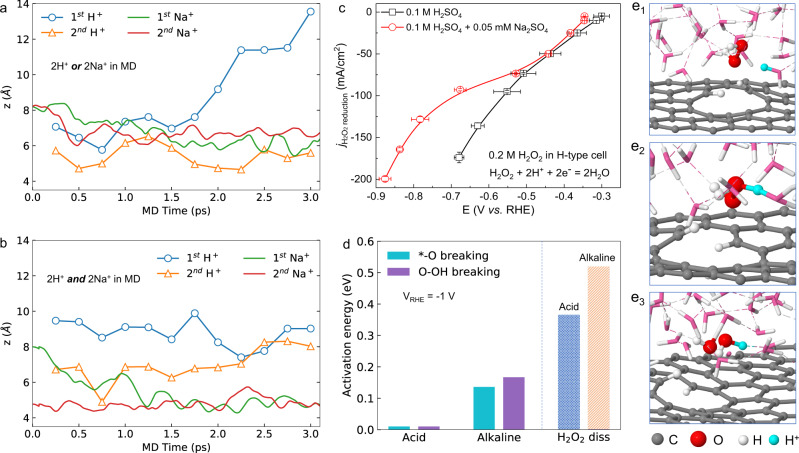
Electrochemical H_2_O_2_ dissociation and simulation of the Na^+^ effect at the local environment of catalyst surface. **a**, **b** Evolution of the position (surface normal direction “z”) of Na^+^ ions (and/or) H^+^ after positioning them in the water layers; **c** the partial current of electrochemical H_2_O_2_ dissociation as a function of the voltage by using 0.1 M H_2_SO_4_ or 0.1 M H_2_SO_4_ + 0.05 M Na_2_SO_4_ electrolyte in H-type cell. The measured potentials were manually 100% compensated. The error bars represent two independent tests. **d** activation energies calculated through constant potential molecular dynamics (see SI for more details). **e**_**1**_–**e**_**3**_, Initial state, transition state and final state of the H_2_O_2_ decomposition in neutral solution. C: grey; O: red; H: white; Proton: cyan.

As shown in Fig. 5a, b, both Na^+^ cations drift towards the surface in molecular dynamics. This is not unexpected considering that under the low potential V_RHE_ = − 1 V, the surface is charged by ~3e^−^. Such a fast drift may have two consequences: firstly, the local concentration of Na^+^ can be much higher than that in the bulk; secondly, as a charge carrier that compensates the net charge of the substrate, Na^+^ may compete with protons which is the major charge carrier in the acid electrolyte when Na^+^ cations are not added. Indeed, as shown in Fig. S17, when Na^+^ cations and protons co-exist near the interface, the cations compete with the protons by repelling the proton away from the surface. After 3 picoseconds of AIMD, both cations stay ~4.5 Å from the surface, while the protons end at ~8 Å from the surface. These results clearly suggest that the cations, which can be enriched by the attraction of the negatively charged surface, can strongly repel the local protons, and thus dramatically reduce the local proton concentrations.

Then we consider how the selectivity of the 2e^−^–ORR is affected by the presence of Na^+^ cations and the reduced local proton concentrations. We understand that carbon catalysts typically bind oxygen intermediates weakly and thus present an intrinsic selectivity towards H_2_O_2_, which can be seen from our above experimental results (high H_2_O_2_ selectivity under low currents in Fig. 2c) as well as previous reports^9,11^. The catalyst’s H_2_O_2_ selectivity starts to drop under significant overpotentials to deliver large currents in pure acids, where two possible reaction mechanisms could play a role in guiding the reaction towards the 4e^−^ pathway of H_2_O. One possibility is that, while the catalyst still produced H_2_O_2_ selectively in the first place, those locally generated H_2_O_2_ under such negative potentials could be further reduced to H_2_O coupling electrons and protons (H_2_O_2_ + 2e^−^ + 2H^+^ = 2H_2_O), resulting in a low apparent H_2_O_2_ selectivity. In this case, as the Na^+^ cation additives can effectively screen away local protons, the further dissociation of generated H_2_O_2_ can be depressed, resulting in better H_2_O_2_ selectivity. This “cation protection” of as-formed H_2_O_2_ can be validated by performing the electrochemical reduction of H_2_O_2_ in both pure and Na^+^-containing acidic electrolytes (Figs. S19–21, Fig. 5c). As shown in Fig. 5c, the H_2_O_2_ reduction activity was greatly suppressed, especially under large overpotentials, when Na^+^ was added, suggesting that the introduction of cations can greatly inhibit the H_2_O_2_ dissociation to H_2_O under reductive potential environments. Please be noted here that the only possible side reaction, the hydrogen evolution reaction, was also taken into consideration when measuring the H_2_O_2_ reduction currents (Fig. S15). This suppression effect can be further validated from our simulation results. Clearly, as shown in Fig. 5d, the absence of protons increases the H_2_O_2_ decomposition barrier to 0.519 eV, 0.156 eV higher than that when a proton is present. The initial state, transition state and final state of H_2_O_2_ decomposition are shown in Fig. 5e and S17. As the O-O bond elongates, the proton attaches to one of the O in H_2_O_2_ and forms a HO-OH_2_ complex at transition state and eventually forms OH^−^ and H_2_O. This process clearly shows how the proton promotes H_2_O_2_ decomposition, explaining the reason why cations could prevent the further reduction of as-synthesized H_2_O_2_ by screening out local protons.

Another possible factor for improved H_2_O_2_ selectivity is that the cations could suppress the dissociation process of peroxide intermediate (*OOH) during ORR due to local proton depletion. To explore this hypothesis, we further used a slow-growth approach based on AIMD to evaluate the reaction barriers of both 2e^−^ and 4e^−^ paths under different proton concentration conditions. As displayed in Fig. 5d, when a proton is present, both the 2e^−^ path and 4e^−^ paths have an extremely low barrier and take place spontaneously at 300 K. In contrast, the absence of protons increases the *-O breaking (2e^−^ path) barrier to 0.136 eV, in comparison to 0.167 eV for O-OH breaking (4e^−^ path), suggesting 2e^−^ pathway being favorable by exp ((0.167–0.136)*k*_B_*T*) = 3.36 times than the 4e^−^ pathway and thus enhanced H_2_O_2_ selectivity. Therefore, the local absence of protons, a result of cation accumulation near the surface, can strongly enhance the H_2_O_2_ selectivity in acids.

We also evaluate the effect of Mg^2+^ on the 2e^−^–ORR by replacing two Na^+^ by one Mg^2+^, and running the AIMD simulations similar to the case of Na^+^ (Fig. 5b). As shown in Fig. S27, Mg^2+^ does not show the same effect as Na^+^. This is likely due to two reasons: (1) Mg^2+^ is more efficiently screened as the Mg^2+^ bonds stronger with O of H_2_O than the case of Na^+^, which is evidenced by the significantly shorter distance between Mg and O (averaged distance *d*_Mg-O_ = 2.05 Å vs. d_Na-O_ = 2.55 Å); (2) the mole concentration of Mg^2+^ is only half of that of Na^+^ in the electrical double layer and the electrostatic repelling decays in the form of 1/*r*, so there is more “screened” space for H^+^ in the electrical double layer.

### Practical generation of H_2_O_2_ using cation exchange membrane solid electrolyte reactor

Obtaining good H_2_O_2_ selectivity and activity in acidic ORR is a prerequisite for practical implementations of membrane electrode assembly (MEA) reactor using reliable and well-established PEM such as Nafion (sulfonated tetrafluoroethylene based fluoropolymer-copolymer membrane). However, till so far, only noble metal catalysts such as PtHg, PdHg or PtP_2_ nanocrystals could deliver reasonable H_2_O_2_ selectivity and stability in proton exchange MEA device^22,23,37^. This observed notable promotion effect of cations on low-cost and non-toxic carbon catalysts in acidic H_2_O_2_ generation therefore provides us with a great opportunity to deliver practical H_2_O_2_ activity, selectivity, and stability. Our basic assumption is that, as the alkali metal cations can move across the PEM, they may help to regulate the local environment of the catalyst/membrane interface for better H_2_O_2_ activity and selectivity. To explore how to successfully make use of this cation tuning effect, we first evaluated its applicability in a traditional PEM-MEA cell configuration (Figs. S22–S24). First, it is well within our expectation that the traditional PEM-MEA cell using commercial carbon black catalyst with 0.1 M H_2_SO_4_ as the anolyte, presented negligible H_2_O_2_ selectivity, due to the high proton flux at the catalyst/membrane interface (Fig. S22). However, we found out that even with the addition of cations in acids (Fig. S23), or directly using Na_2_SO_4_ solution as the anolyte (Fig. S24), no obvious improvements were observed. This is because on the anode side, a significant number of protons will be generated locally at the catalyst/membrane interface during the oxygen evolution reaction and then immediately transported across the membrane to the cathode side, suppressing the possibility of Na^+^ ion transportation from the bulk anolyte towards the cathode to regulate the interfacial environment of improved 2e^−^–ORR. The low H_2_O_2_ selectivity in PEM-MEA drove us to design a new cell configuration to employ this cation effect for continuous production of H_2_O_2_ in a practical way.

**Fig. 6:**
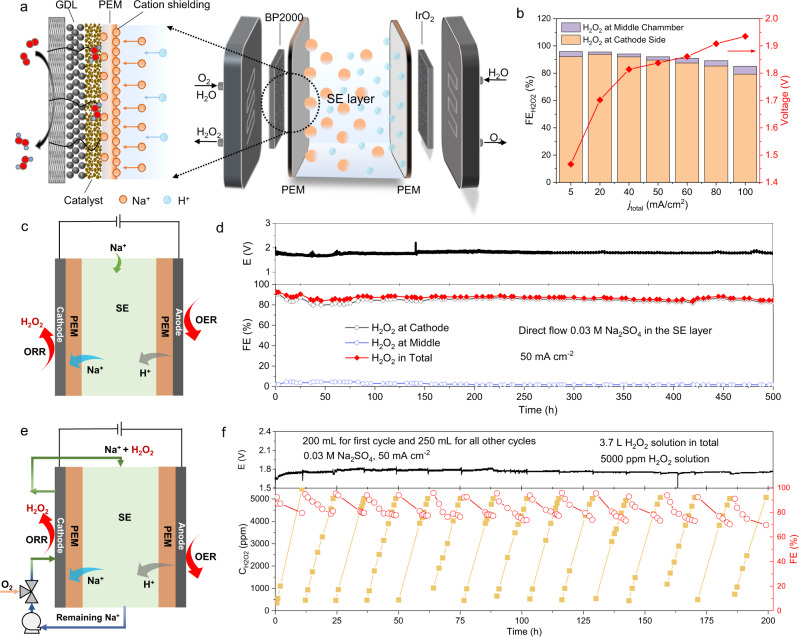
Continuous production of H_2_O_2_ solution using carbon catalyst in a SE cell with a double-PEM configuration. **a** Schematic illustration of reducing O_2_ to H_2_O_2_ in our SE cell with double-PEM configuration. The O_2_ + H_2_O/PEM//SE//PEM/H_2_O cell in which O_2_ is reduced at the cathode side to form H_2_O_2_ and flowed out by H_2_O flow. The cations in the middle chamber cross over the PEM under an applied reduction potential and move to the cathode, protecting the catalyst surface for the production of H_2_O_2_. **b** The I–V curve and corresponding FEs for producing H_2_O_2_ using the SE cell with double-PEM configuration through flowing 0.03 M Na_2_SO_4_ in the middle chamber. The concentration of Na_2_SO_4_ can be varied. **c**, **d** The schematic illustration and chronopotentiometry stability test of the SE cell with double-PEM configuration by directly flowing 0.03 M Na_2_SO_4_ solution in the middle chamber at 50 mA·cm^−2^ current density. The flow rate of Na_2_SO_4_ solution is 2.7 mL min^−1^. The oxygen gas (flow rate 180 sccm) and DI water (flow rate 10.8 mL min^−1^) are mixed and flowed into the cathode to producing H_2_O_2_ solution. DI water with flow rate of 2.7 mL min^−1^ was circulated at the anode side. **e**, **f** The schematic illustration and chronopotentiometry stability test of the SE cell for practically producing 5000 ppm H_2_O_2_ solution. The volume of Na_2_SO_4_ stock solution in the first cycle is 200 mL, and the other 14 cycles hold 250 mL. The SE cell can produce around 3.7 L 5000 ppm H_2_O_2_ solution in 15 cycles for more than 200 h. The liquid flow rate is 4.5 mL min^−1^ and the O_2_ gas flow rate is 140 sccm. The total geometric area of the flow field in the cathode of our SE cell is 4 cm^2^.

Instead of the traditional PEM-MEA cell design, here we developed a SE reactor with three chambers separated by two PEMs to fully implement this cation effect for high-performance H_2_O_2_ generation (Fig. 6a and Fig. S25). Specifically, the cathode (carbon black) and anode (IrO_2_) of our device are catalyst-coated GDL electrodes, which were separated by a thin SE layer sandwiched by two identical PEMs (Nafion-117). The cathode side was continuously supplied with a mixture of O_2_ stream and water flow for 2e^−^–ORR, while the anode side was circulated with H_2_O for water oxidation. In the middle chamber, a SE layer consisting of porous polymer ion conductors was used to minimize the *iR*-drop between cathode and anode^38,39^. A dilute cation solution flows through this SE layer to introduce the cation effects on the cathode side 2e^−^–ORR. Please be noted here that without this SE layer, the cell voltage was significantly increased due to the increased cell resistance between the cathode and anode (Fig. S26). Under a negative reduction potential, the cations in the middle SE chamber are driven by the electrical field to penetrate the PEM toward the cathode surface and thus regulate the local environment at the catalyst/PEM interface to promote H_2_O_2_ generation (Supplementary Note 2 and Fig. S27). The H_2_O_2_ molecules formed at the cathode side are then efficiently brought out via the oxygen and DI water flow stream. Meanwhile, protons generated from water oxidation at the anode penetrate the righthand side PEM and move into the middle chamber to compensate for the charge.

The I–V curve of our 4-cm^2^ three-chamber PEM SE cell flowing 0.03 M Na_2_SO_4_ solution in the middle layer is plotted in Fig. 6b. Of note that the concentration of Na_2_SO_4_ can be varied to higher values. Our target is to realize the high production rate of H_2_O_2_ while minimizing cations’ consumption for practical demonstrations. Therefore, a 0.03 M Na_2_SO_4_ solution is adopted to regulate the interfacial environment of improved 2e^−^–ORR in our SE cell. A mixture of O_2_ gas flow (180 sccm) and DI water flow (1.8 mL·min^−1^) was supplied to the cathode. The flow of DI water is to efficiently bring out the generated H_2_O_2_. By ramping up the overall current density, the cell voltage of the SE reactor gradually increased. The H_2_O_2_ FE remained over 85% across the entire cell voltage range, with a maximum of 96% at 5 and 20 mA cm^−2^ (Fig. 6b), much higher than the traditional MEA cell configuration. In comparison, the reactor without alkali metal cations requires a higher potential to deliver and shows much lower H_2_O_2_ FE, practically at high current densities (Fig. S28). The crosse-over Na^+^ from the middle SE layer to the cathode plays a key role in determining the H_2_O_2_ FE (Fig. S27).

The electrolysis stability is always one of the most important but challenging parts in practical applications. Benefiting from the stable material properties in carbon black and PEM as well as reliable cation effects, our SE cell with the double-PEM configuration presents excellent long-term stability in producing H_2_O_2_. The SE reactor stability was evaluated by holding a 50 mA·cm^−2^ cell current density (200 mA total current). As shown in Fig. 6c and d, when supplying dilute Na^+^ ions in the SE layer_,_ a continuous generation of H_2_O_2_ solution on the cathode side can be stably operated for over 500 h with no degradations in product FE (~90%). As a sharp contrast, in the absence of cations, the H_2_O_2_ FE started with less than 60% and rapidly dropped to less than 10% in 6 h, which can even be recovered to ~90% when cations were later introduced (Fig. S29), clearly suggesting this prominent cation effect. The observation indicates that the carbon catalyst still works well, and this is not the reason for FE degradation. We suppose the FE degradation is because of the accumulation of local H^+^ to high concentrations with the extension of reaction time, which accelerates the further reduction of H_2_O_2_ to H_2_O and decrease the H_2_O_2_ selectivity.

A continuous supply of cation solutions in the SE layer could limit the device’s real applications due to the following two reasons. First, it could result in a significant consumption of cations as most of them are flowing out of the SE layer and only part of them crossed to the cathode chamber. Second, the generated H_2_O_2_ solution in the cathode would be slightly alkaline due to the Na^+^ ion crossover from the SE layer (resulting in slight acidity in the SE layer downstream flow). To further explore the high potential of our SE cell for practical uses, we operated the cell by circulating the cation solution from the SE layer into the cathode side, and then back to the SE layer for H_2_O_2_ accumulation. The outlet solution in the middle chamber was mixed with oxygen flow and supplied to the cathode to produce H_2_O_2_. Then the as-produced solution containing H_2_O_2_ and cations was cycled back into the middle chamber (Fig. 6e). By doing so we could continuously reuse the cations by circulating them back to the SE layer for a closed system without the need for a continuous cation stream supply. Also, the excessive OH^−^ groups generated from the cathode would be neutralized by exactly the same number of excessive protons in the SE layer. Our target is to accumulate the H_2_O_2_ concentration to ~5000 ppm in a 250 mL solution (containing only 60 mM of Na^+^) for each operation cycle via maintaining a 50 mA cm^−2^ cell current. As shown in Fig. 6f, the H_2_O_2_ concentration continuously increased to ~5000 ppm in about 13 h. The cell can be operated for more than 200 h with negligible degradations. During this stability test, a total of 3.7 L 5000 ppm H_2_O_2_ solution was obtained. We also observed that, in each operation cycle the H_2_O_2_ FE slightly decreases with increased H_2_O_2_ concentration, which may be due to the H_2_O_2_ self-decomposition, further reductions on the cathode, and/or the crossover oxidation on the anode side. For each operation cycle, the H_2_O_2_ concentration can reach up to ~0.15 M, which is 5 times of the Na_2_SO_4_ additive in the final product. A higher concentration of H_2_O_2_ solutions can also be produced by extending the operation time while maintaining the current and FE. As a result, a high concentration of 20,000 ppm H_2_O_2_ solution was achieved in 17 h by circulation of the 50 mL water at the cathode side (Fig. S30). Based on the above promising H_2_O_2_ activity, selectivity and especially the durability, and since all the reactor components, including catalysts, membrane, and the polymer SE are all commercially available, our PEM-based H_2_O_2_ SE cell with cation promotion effect has a great potential for future’s practical applications.

To conclude, we presented a cation-regulated interfacial engineering approach to improve the catalytic performance of O_2_ reduction to H_2_O_2_ at industrial-relevant rates in strong acidic media. By adding a small number of alkali metal cations in acid solutions, the selectivity and stability of H_2_O_2_ generation using commercial carbon black catalyst can be dramatically improved, especially under large ORR current densities (over 400 mA cm^−2^). Modeling of reaction and local environment suggest that the cations could preferentially be attracted to the catalyst/electrolyte interface, showing a “shielding effect” to squeeze out the catalyst/electrolyte interfacial protons and thus prevent further reduction of generated H_2_O_2_ to water. A double-PEM-based reactor was further developed for continuous production of H_2_O_2_ solution. By using only 0.03 M Na_2_SO_4_ as the cation source, a promoted H_2_O_2_ FE (~90%) and stability (>500 h) were achieved. In light of this performance, this would be a promising demonstration of the use of renewable electricity for the continuous generation of H_2_O_2_ through O_2_ reduction at a more practical scale. This cation “shielding effect” could also be used in other electrocatalytic reactions such as selective CO_2_ reduction into fuels and chemicals or N_2_ reduction into ammonia.

### Experiment

#### Materials

All chemicals including lithium sulfate (Li_2_SO_4_), sodium sulfate (Na_2_SO_4_), potassium sulfate (K_2_SO_4_), caesium sulfate (Cs_2_SO_4_), perchloric acid, sulfuric acid, and Nafion perfluorinated resin solution (527084-25 mL) were purchased from Sigma Aldrich. H_2_O_2_ solution (35 wt%) was purchased from Merck & Co. Vulcan XC-72 were purchased from Fuel Cell Store. The conductive carbon black BP2000 was purchased from Cabot Corporation. Millipore water (18.2 MΩ·cm) was used throughout all experiments.

#### Preparation of electrodes

Typically, 40 mg conductive carbon black (BP2000) and 80 μL of Nafion (527084-25 mL) binder solution was mixed with 4 mL of 2-proponal (Sigma-Aldrich) and 1 ml methanol. After sonication in ice water for 30 min, the obtained homogeneous ink was air-brushed onto a 5 × 5-cm^2^ gas diffusion layer (GDL, Sigracet 28 BC, Fuel Cell Store) electrode at room temperature. Then the prepared electrode was dried in a vacuum at room temperature for 24 h before use. The procedure for preparation electrodes with other catalysts is same as that of carbon black BP2000. The reduced graphene oxide (rGO) catalyst was pretreated using HCl and acetone to remove impurities before making the catalyst ink.

#### Activation of the Nafion-117 membrane

The proton exchange membrane (PEM, Nafion-117) was purchased from Fuel Cell Store. The Nafion-117 membrane was pre-treated with 5% (v/v) H_2_O_2_ for 1 h at 80 °C and 10% (v/v) H_2_SO_4_ for 1 h at 80 °C before assembling a cell.

#### Materials characterization

The scanning electron microscopy (SEM) was performed on an FEI Quanta 400 field emission scanning electron microscope. BET surface area analysis was performed using Quantachrome Autosorb-iQMP/Kr BET Surface Analyzer.

#### Electrochemical test in flow cell

The electrochemical H_2_O_2_ generation was conducted at 25 °C by using a conventional flow cell with a typical three-electrode setup, and the electrochemical response was recorded by using a BioLogic VMP3 workstation. The cathode and IrO_2_ anode (Fuel Cell Store) were placed on opposite sides of two 0.5 cm thick PTFE plates with 0.5 cm * 2 cm channels. The catalyst layers faced the flowing liquid electrolyte, and the geometric surface area of the catalyst was 1 cm^2^. A Nafion-117 film was sandwiched by the two PTFE plates to separate the chambers. At the cathode side, 30 sccm humidified O_2_ was supplied through a titanium gas flow chamber, and a catholyte containing 0.1 M H_2_SO_4_ and cations flowed into the cathode chamber. The catholyte flow rate of 1.8 mL min^−1^ was controlled by a syringe pump. The pH value of the catholyte was determined by an Orion 320 PerpHecT LogR Meter (Thermo Scientific). At the anode side, 0.1 M H_2_SO_4_ anolyte was circulated with a flow rate of 1.8 mL min^−1^ for O_2_ evolution reaction as the counter electrolyte. A saturated calomel electrode (SCE, CH Instruments) was employed as the reference electrode. All potentials measured against SCE were converted to the reversible hydrogen electrode (RHE) scale using E_RHE_ = E_SCE_ + 0.241 V + 0.0591 × pH. The resistance (Rs) of the catalytic system was determined by potentiostatic electrochemical impedance spectroscopy (PEIS) at frequencies ranging from 0.1 Hz to 200 kHz. All the measured potentials using the three-electrode flow cell setup were manually 85% compensated unless stated otherwise.

#### Solid state electrolyte cell with double-PEM configuration

The continuous electrosynthesis of H_2_O_2_ was conducted using a solid electrolyte (SE) cell with a sandwiched double-PEM configuration. The cell configurations and the production setup are illustrated in Fig. 6a and Fig.  S25. The cathode side was supplied with an oxygen/water mixture of 180 sccm of O_2_ gas and 10.8 mL min^−1^ of DI water. The gas flow rate was controlled by a mass flow meter (MFC) and the water flow rate was controlled by a syringe pump. The flow rate of H_2_O_2_ product at the outlet was calibrated using a measuring cylinder. The fast water flow in the gas/liquid mixture through the cathode chamber is beneficial for bringing out the generated H_2_O_2_ molecules and decreasing the further electroreduction of H_2_O_2_. In the middle chamber, the styrene-divinylbenzene sulfonated copolymer Dowex 50WX8 hydrogen form (Sigma–Aldrich) cation conductor was employed as the SE. A solution containing H_2_SO_4_ and/or Na_2_SO_4_ flowed into the SE layer controlled by a syringe pump. The anode side was circulated with 0.1 M H_2_SO_4_ at 2.7 mL min^−1^. All the measured potentials using a two-electrode setup were manually 100% compensated unless stated otherwise.

#### Bath synthesis of 5000 ppm H_2_O_2_ Solution using double-PEM cell

The bath electrosynthesis of 5000 ppm H_2_O_2_ was conducted using double-PEM cell configuration (as shown in Figs. 6a and S25). A certain volume of 0.03 M Na_2_SO_4_ solution (200 mL for the first cycle, and 250 mL for other cycles) was supplied into the middle SE layer with flow rate of 4.5 ml min^−1^. The outlet of the middle chamber was mixed with 140 sccm O_2_ gas and then supplied to the cathode side for producing H_2_O_2_. The cathode outlet containing H_2_O_2_ and remaining Na_2_SO_4_ was then circulated back to the middle SE chamber for continually running of the cell. Once the accumulated H_2_O_2_ concentration reached around 5000 ppm, the cell was flushed with fresh 0.03 M Na_2_SO_4_ for 10 min to remove residue H_2_O_2_, and another bottle of fresh 0.03 M Na_2_SO_4_ (250 mL) was used to start a new batch.

#### Determination of the H_2_O_2_ concentration

The concentration of the generated H_2_O_2_ was determined through a titration process. After electrolysis, the as-produced H_2_O_2_ solution was collected and evaluated using the standard potassium permanganate (0.1 N KMnO_4_ solution, Sigma–Aldrich) titration process, according to the following equation:

1 $$2{{{{{{{\rm{MnO}}}}}}}_{4}}^{-}+5{{{{{{\rm{H}}}}}}}_{2}{{{{{{\rm{O}}}}}}}_{2}+6{{{{{{\rm{H}}}}}}}^{+}\to 2{{{{{{\rm{Mn}}}}}}}^{2+}+5{{{{{{\rm{O}}}}}}}_{2}+8{{{{{{\rm{H}}}}}}}_{2}{{{{{\rm{O}}}}}}$$

The sulfuric acid (1 M H_2_SO_4_) was used as the H^+^ source. The FE for H_2_O_2_ production is calculated using the following equation:

2 $${{{{{\rm{FE}}}}}}=\; 	\frac{{{{{{\rm{generated}}}}}}\,{{{{{{\rm{H}}}}}}}_{2}{{{{{{\rm{O}}}}}}}_{2}\left({{{{{\rm{mol}}}}}}\,{{{{{{\rm{L}}}}}}}^{-1}\right)\times 2\times 96485\;\left({{{{{\rm{C}}}}}}\,{{{{{{\rm{mol}}}}}}}^{-1}\right)\times {{{{{\rm{flow}}}}}}\,{{{{{\rm{rate}}}}}}\;({{{{{\rm{mL}}}}}}\,{{{{{{\rm{s}}}}}}}^{-1})}{{j}_{{{{{{\rm{total}}}}}}}({{{{{\rm{mA}}}}}})}\\ 	\times 100\;({{{{{\rm{maximum}}}}}}\,100 \% )$$

#### Electrochemical H_2_O_2_ dissociation

The electrochemical H_2_O_2_ dissociation was conducted in a customized gas-tight H-type glass cell at 25 °C. Before the experiment, the glass cell was carefully cleaned by boiling the cell in a mixture of H_2_SO_4_: H_2_O_2_ (3:1) for 1 h. After being thoroughly cleaned by DI at room temperature, the cell was further boiled in DI water for another 1 h to totally remove H_2_O_2_ residual.

The electrochemical H_2_O_2_ dissociation was conducted with a BioLogic VMP3 workstation. The cathode electrode was prepared by spray coating carbon black (BP2000) on a GDL (Sigracet 28 BC, Fuel Cell Store), and the anode electrode was a carbon rod. The cathode electrode was fixed using a gold-coated clip and the exposed geometric surface area of each electrode was 1 cm^2^. Since the traditional clip made of iron can be easily dissolved to Fe^2+^/Fe^3+^ by acid and may contribute to the H_2_O_2_ dissociation, the gold-coated clip is necessary to avoid the dissolution of the iron clip during the process. The working and counter electrodes were parallel and separated by a clean PEM. The mixture of 0.2 M H_2_O_2_ + 0.1 M H_2_SO_4_ was used as catholyte, 0.1 M H_2_SO_4_ was used as anolyte, and each volume of the electrolyte was 25 mL. A gas dispersion frit was used at the cathode chamber to provide vigorous electrolyte mixing. The cathode chamber was supplied with Ar gas (99.999% Praxair) at a rate of 20 sccm for 30 min before the electrochemical measurements. During electrolysis, continuous Ar flow was supplied throughout the experiment and the gas outlet was connected to a gas chromatograph (GC, Shimadzu GC-2014 GC) for detection of the H_2_ gas. The H_2_ amount was quantified by a thermal conductivity detector. After the electrochemical decomposition, the amount of H_2_O_2_ remaining was determined by using the standard potassium permanganate titration process.

#### Theoretical simulation

The Vienna Ab initio Simulation Package (VASP)^40,41^, together with the VASPsol patch^42^, was employed to perform slow-growth calculation. The constant potential along the MD track is realized by adjusting the number of electrons on-the-fly, as described in our previous work^35^. Perdew-Burke-Ernzerhof (PBE) functional^43^ together with D3 van der Waals correction^44^ were employed in most of the calculations. The cutoff energy of the plane-wave basis is 400 eV in the relaxation while 300 eV was used in the MD simulations. Gamma-only MD calculations were done for the thick model in Fig. 5a, b (water layers equivalent to 6 ice layers), while 3 × 3 × 1 Gamma-centered k-mesh was used in MD simulations using the thinner defect-graphene model shown in Fig. S17. One proton is added into the 45 H_2_O molecules to simulate the pH = 0 condition. We choose the bond length (*-O or O-OH) to be the reaction coordinate (*ε*). Time step in MD was set to be 0.5 femtoseconds, and the ∂*ε* in slow-growth method^35^ was set to be 0.0004 Å. A Nose-Hoover thermostat^45^ was used to keep temperature (statistically) constant at 300 K. The proton is determined as the H that is farthest from the central O among two other H atoms of a H_3_O^+^ for snapshots that are evenly distributed along the AIMD track.

## Supplementary information

Supporting Information

Peer review file

## Data Availability

The data that support the findings of this study are available from the corresponding authors upon reasonable request.
