# Supplementary material for: Electrochemical oxygen reduction to hydrogen peroxide at practical rates in strong acidic media
Source: Nat Commun. 2022 May 24;13:2880. doi: 10.1038/s41467-022-30337-0 (PMC9130276; doi:10.1038/s41467-022-30337-0)
Supplement: Supplementary file 1 — Supporting Information [file 41467_2022_30337_MOESM1_ESM.pdf]

Supporting Information

Electrochemical Oxygen Reduction to Hydrogen Peroxide at Practical Rates in Strong Acidic Media

Xiao Zhang[1]†*, Xunhua Zhao[2]†, Peng Zhu[1], Zachary Adler[1], Zhen-Yu Wu[1], Yuanyue Liu[2*], Haotian Wang[1,3,4]*

[1]Department of Chemical and Biomolecular Engineering, Rice University, Houston, TX 77005, USA.

[2]Texas Materials Institute and Department of Mechanical Engineering, The university of Texas at Austin, Austin, Texas 78712, USA.

[3]Department of Chemistry, Rice University, Houston, TX 77005, USA.

[4]Department of Materials Science and NanoEngineering, Rice University, Houston, TX 77005, USA.

†These authors contributed equally.

*Corresponding author Email: htwang@rice.edu (H.W.), yuanyue.liu@austin.utexas.edu (Y.L.) and xiao1.zhang@polyu.edu.hk (X. Zhang).

Supplementary Note 1:

The effect of alkali metal cations toward $4e^-$-ORR on Pt and carbon catalysts have been previously studied in different pH electrolytes through rotating ring-disk electrode (RRDE)[1-4]. We believe the local environment at the catalyst surface in RRDE does not reflect the real reaction condition. First, RRDE is usually operated with strong agitation, which could disturb the alkali metal cation/proton distribution in the near-electrode layer, especially when the agitation force is stronger than the electrostatic force between catalysts and cations. Second, the steady-state surface concentration of H_2O_2 near the electrode of RRDE is low due to efficient electrolyte flush to the electrode surface, thus the further H_2O_2 reduction could be minimized, which is different from that of practical operation[5,6]

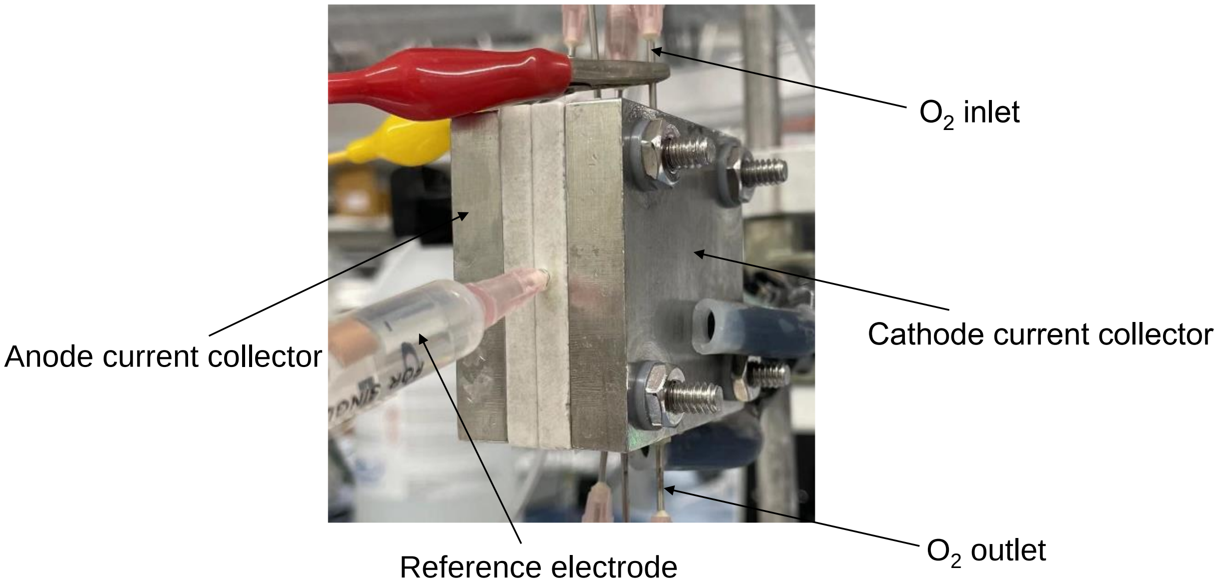

Figure S1. **Photo of a flow cell electrolyzer**. The two outmost titanium plates serve as current collectors. Two PTFE plates, separated by a Nafion-117 PEM, are placed in the middle as an electrode holder and flow channel of electrolyte with a channel size of 0.5 cm × 2 cm. The effective geometric electrode area is confined to be 1 cm^2. A saturated calomel reference electrode (SCE) is connected to the cathode channel. The gas flow rate is controlled to be 30 sccm by a mass flow meter (MFC) at cathode side and the liquid flow rate in the anode side is maintained at 1.8 mL min^{-1} by syringe pumps.

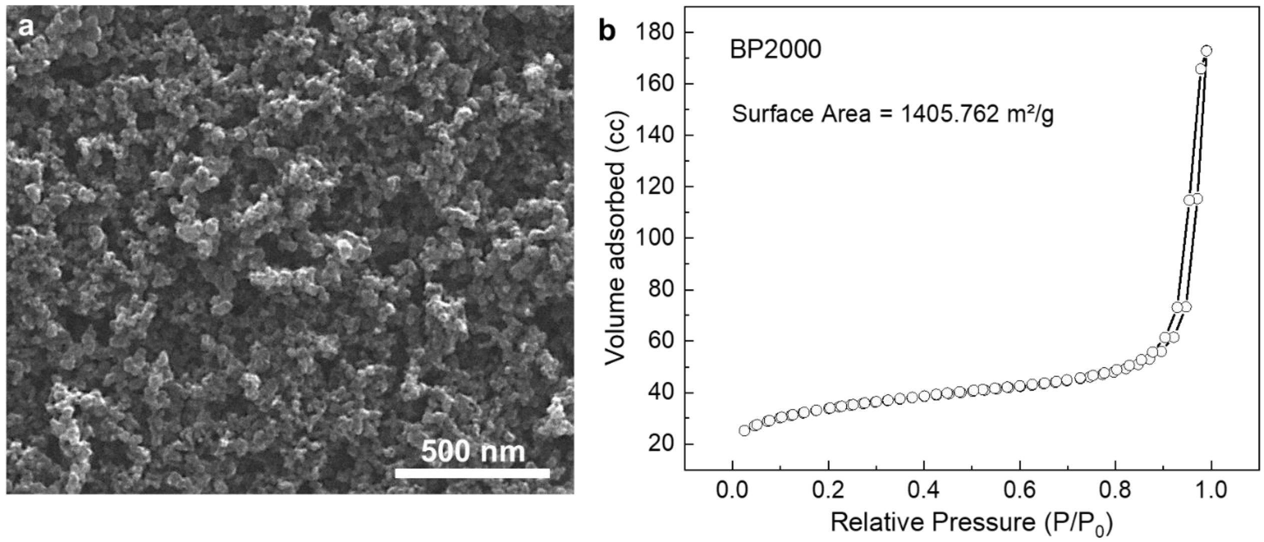

Figure S2. **Characterization of conductive carbon-black BP2000 catalyst**. (**a**) SEM image and (**b**) BET surface area analysis of BP2000 catalyst. The results show that BP2000 has a high surface area (1405.762 m^2/g) and the catalyst layer on the GDL is highly porous, both of which are beneficial for O_2 diffusion during the oxygen reduction reaction (ORR) process.

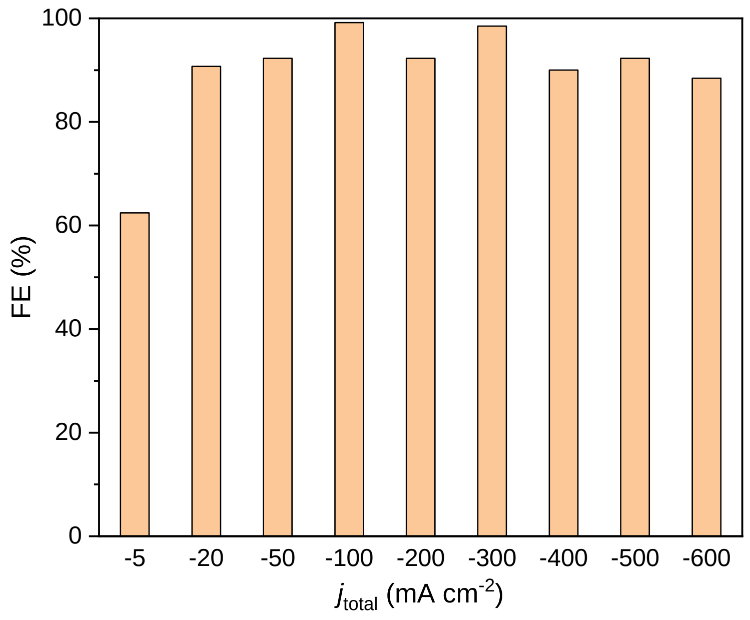

Figure S3. FE of H_2O_2 production through $2e^-$-ORR in a flow cell using 0.1 M H_2SO_4 + 0.2 M Na_2SO_4 as catholyte.

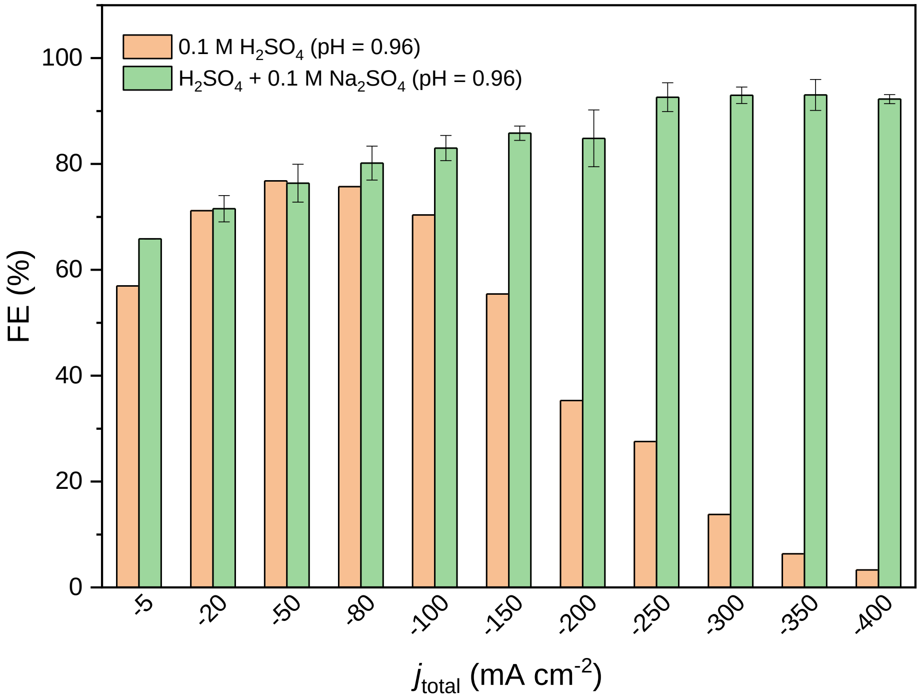

Figure S4. The Na^+ effect toward the H_2O_2 selectivity by using H_2SO_4 or H_2SO_4 + 0.1 M Na_2SO_4 with the same pH value as electrolyte. The pH of the 0.1 M H_2SO_4 + 0.1 M Na_2SO_4 (original pH = 1.13) was adjusted to be same as 0.1 M H_2SO_4 (pH = 0.96) by using concentrated H_2SO_4. Even though the two solutions have the same pH value, the H_2O_2 selectivity using Na^+ as additive is still much higher than that of neat H_2SO_4, indicating that the Na^+ additive, rather than the bulk solution pH, play a key role in determining the H_2O_2 selectivity. The Na^+ cations are supposed to modify the local environment of the catalyst surface during ORR.

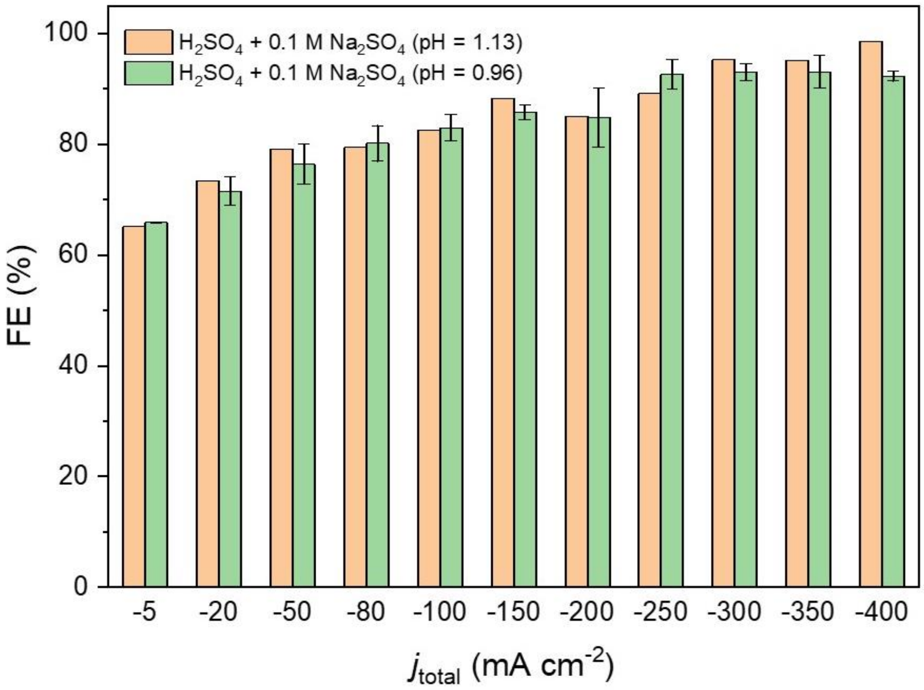

Figure S5. The pH effect of bulk solution toward the selectivity of H_2O_2 production. Two kinds of electrolytes containing H_2SO_4 + 0.1 M Na_2SO_4 with different pH were evaluated. The error bars represent two independent tests. For 0.1 M H_2SO_4 and 0.1 M H_2SO_4 + 0.1 M Na_2SO_4, the pH value is 0.96 and 1.13, respectively. One of the 0.1 M H_2SO_4 + 0.1 M Na_2SO_4 electrolyte was adjusted to give a pH value of 0.96, same as 0.1 M H_2SO_4 (pH = 0.96), by using concentrated H_2SO_4. Both electrolytes have the same Na^+ concentration but different pH of bulk solution. The similar H_2O_2 FE using the two electrolytes indicates that the slightly pH changes of the bulk solution by salt additives (e.g., Na_2SO_4) in our work has negligible effect on the selectivity of electrogenerated H_2O_2 through $2e^-$-ORR.

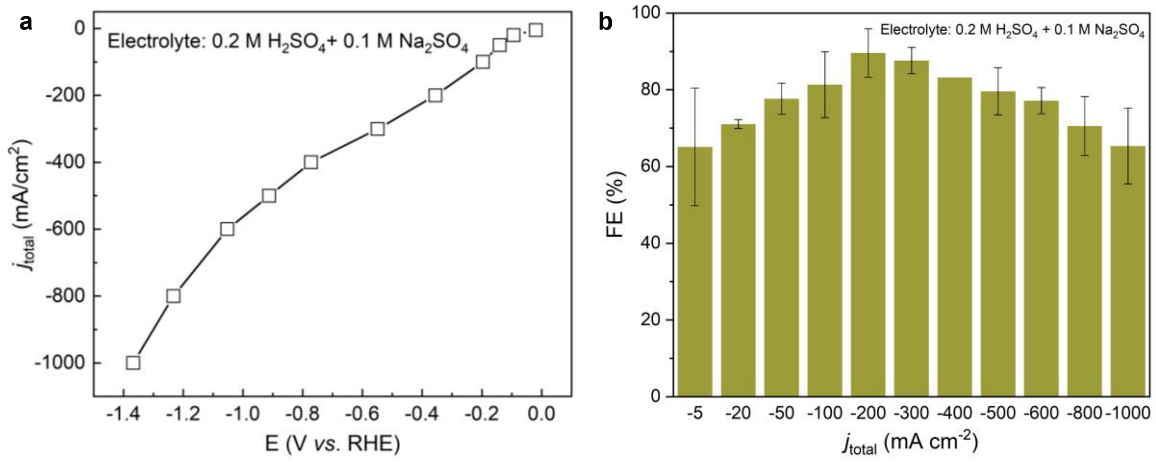

Figure S6: (**a**) The I-V curve and (**b**) corresponding FE of H_2O_2 production through $2e^-$-ORR in a flow cell by using 0.2 M H_2SO_4 + 0.1 M Na_2SO_4 as catholyte. With the presence of 0.1 M Na_2SO_4 as additive, the carbon catalyst BP2000 can drive the $2e^-$-ORR up to 1000 mA cm^{-2} while maintaining 65% H_2O_2 selectivity. The error bars represent two independent tests.

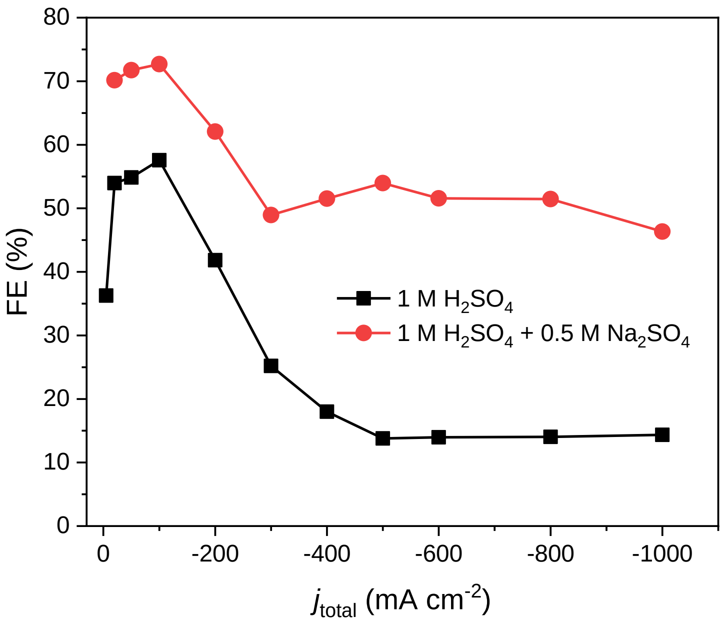

Figure S7. FE of H_2O_2 production through $2e^-$-ORR in a flow cell by using 1 M H_2SO_4 + 0.5 M Na_2SO_4 as catholyte. The H_2O_2 selectivity of carbon black BP 2000 catalyst at 1 M H_2SO_4 shows relatively good (~70%) selectivity under small current regions, but decreases dramatically at high current densities, reaching only ~15 % at 1,000 mA cm^{-2}. However, when we introduced 0.5 M Na_2SO_4 additive in 1 M H_2SO_4 as the electrolyte, the H_2O_2 selectivity was significantly improved, especially under high current densities. The Na^+ additive helped the carbon black catalyst to hold a high H_2O_2 selectivity plateau of ~ 50% until 1,000 mA cm^{-2}, suggesting a more than triple FE compared to that in pure 1 M H_2SO_4. The above observation is similar to what we observed using 0.1 M H_2SO_4 as electrolyte, indicating this is a general phenomenon by using cations to promote the H_2O_2 production in strong acidic conditions.

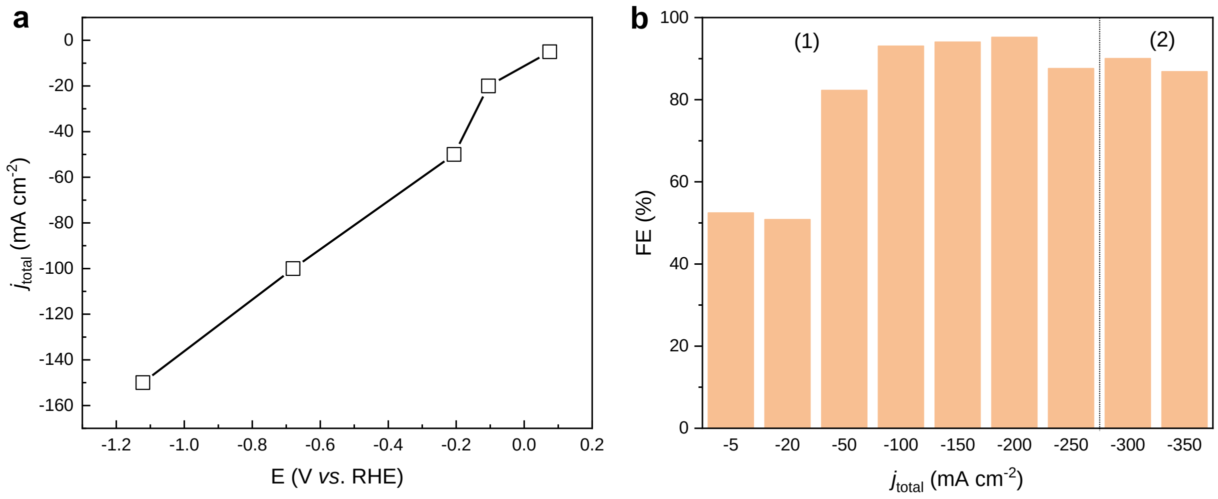

Figure S8. The influence of Na^+ at anolyte on the H_2O_2 production through ORR in a flow cell. (**a**) The I-V curve and (**b**) corresponding FE of H_2O_2 in flow cell by using anolyte containing Na^+ (0.1 M Na_2SO_4 or 0.1 M H_2SO_4 + 0.1 M Na_2SO_4). The (1) in Fig. S8b indicate the anolyte is 0.1 M Na_2SO_4, and (2) in Fig. S8b indicate the anolyte is 0.1 M H_2SO_4 + 0.1 M Na_2SO_4.

For the traditional electrocatalytic ORR process using flow cell configuration, Na_2SO_4 is widely used as the anolyte to balance the electrochemical reaction. However, we found that the Na^+ in the anolyte can easily penetrate the PEM and move to the cathode in the flow cell, greatly influencing the ORR performance at the cathode. The cross-over Na^+ can greatly improve the $2e^-$ selectivity for producing H_2O_2 while inhibiting the $4e^-$ process for producing H_2O. Therefore, for evaluation of the performance of the ORR process through flow cell, it is suggested to use the same solution at anode and cathode during the electrolysis.

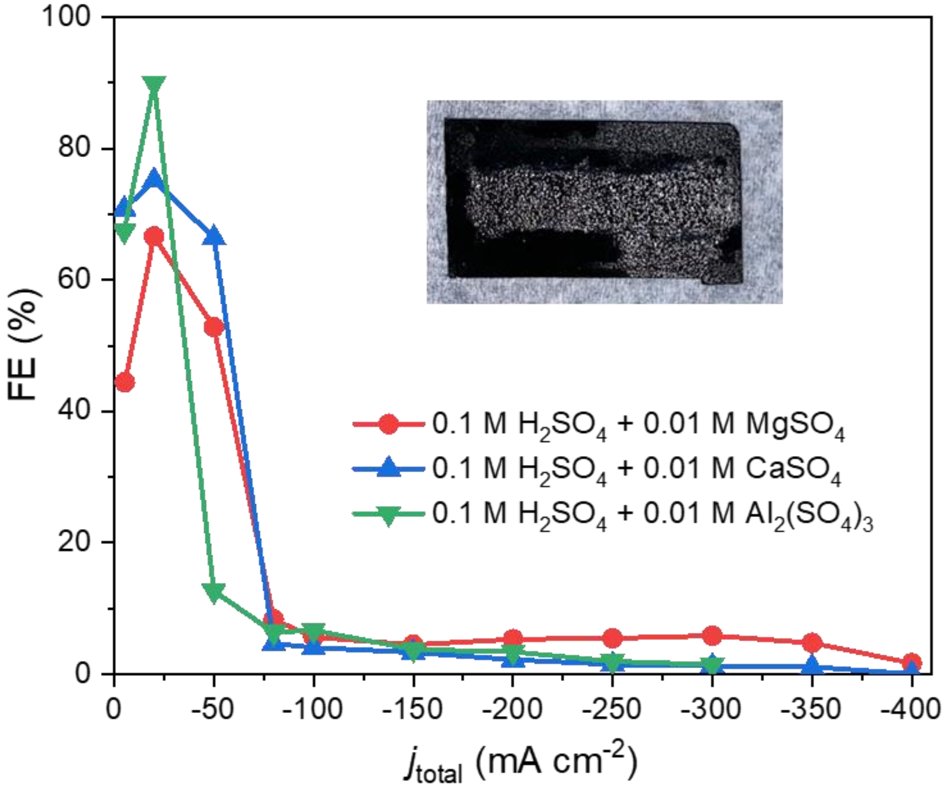

Figure S9. The FEs of H_2O_2 production through 2e$^-$-ORR in a flow cell by using 0.1 M H_2SO_4 + 0.01 M $MgSO_4$, 0.1 M H_2SO_4 + 0.01 M $CaSO_4$, 0.1 M H_2SO_4 + 0.01 M $Al_2(SO_4)_3$ as catholyte, respectively. The inset picture is the carbon electrode after testing using 0.1 M H_2SO_4 + 0.01 M $MgSO_4$ as electrolyte. The white powders are deposited on the surface of the electrode.

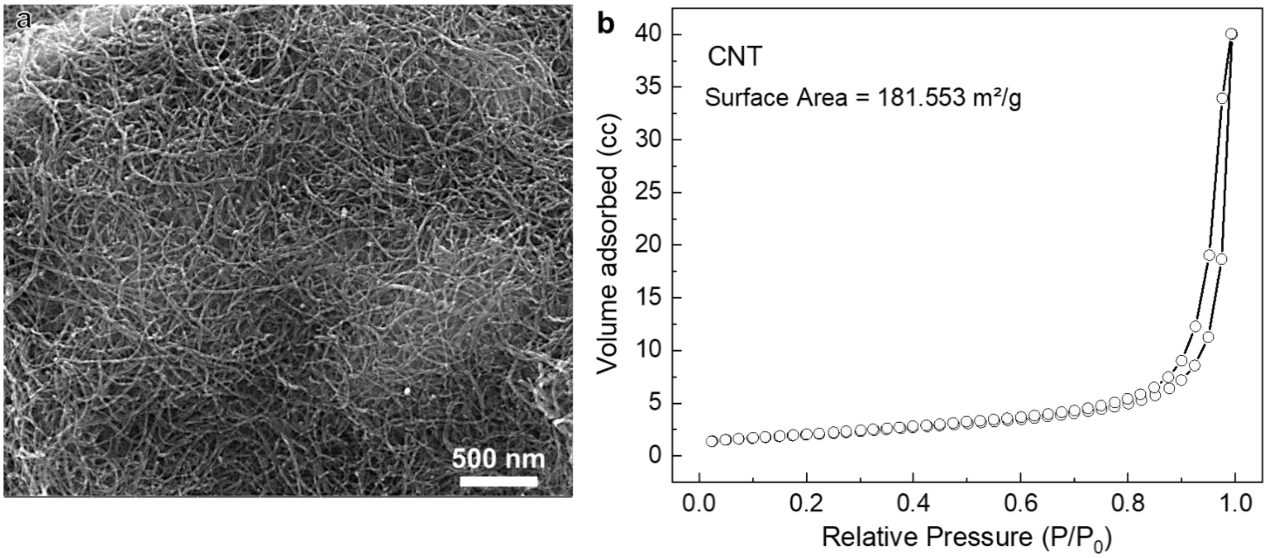

Figure S10. Characterization of carbon nanotube (CNT) catalyst. (**a**) SEM image and (**b**) BET surface area analysis of CNT.

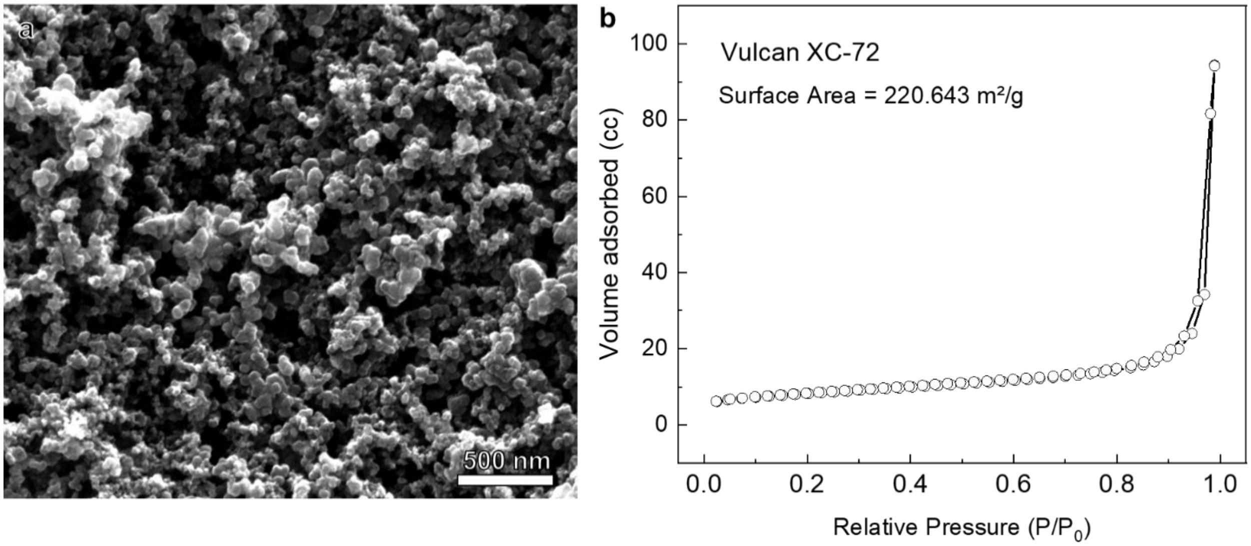

Figure S11. Characterization of Vulcan XC-72 catalyst. (**a**) SEM image and (**b**) BET surface area analysis of Vulcan XC-72 catalyst.

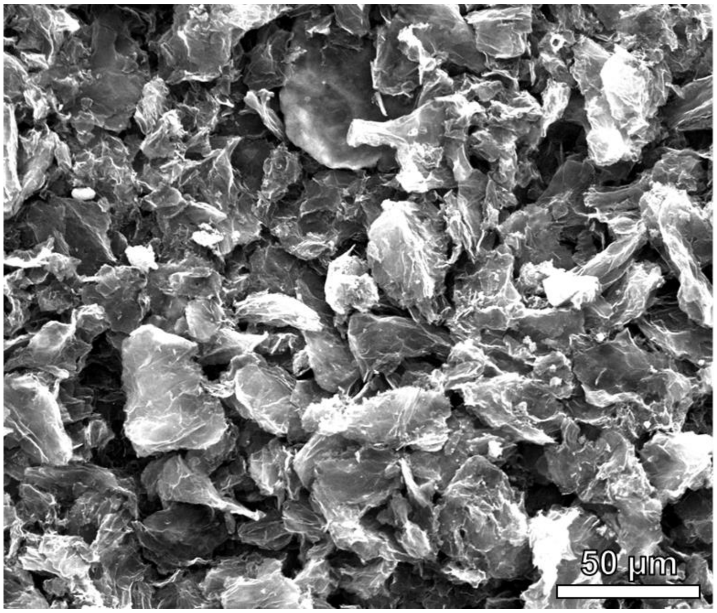

Figure S12. SEM image of rGO catalyst on GDL.

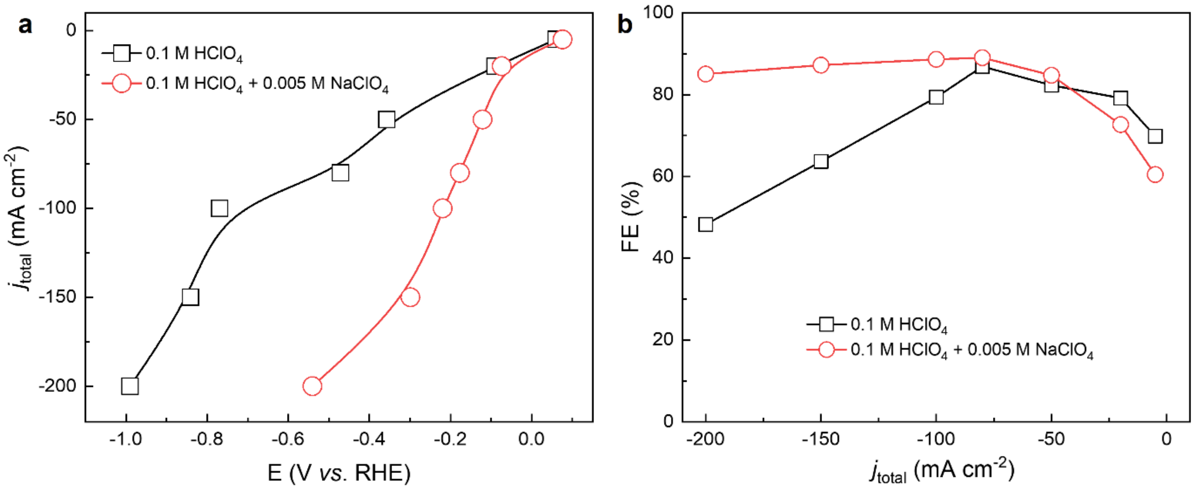

Figure S13. (**a**) The I-V curve and (**b**) FE of H_2O_2 production through 2e$^-$-ORR in 0.1 M $HClO_4$ with/without 0.005 M $NaClO_4$. The Na^+ effect toward 2e$^-$-ORR in $HClO_4$ show a similar trend with that in H_2SO_4, indicating the broad applicability of alkali metal cations for producing H_2O_2 in acid through ORR.

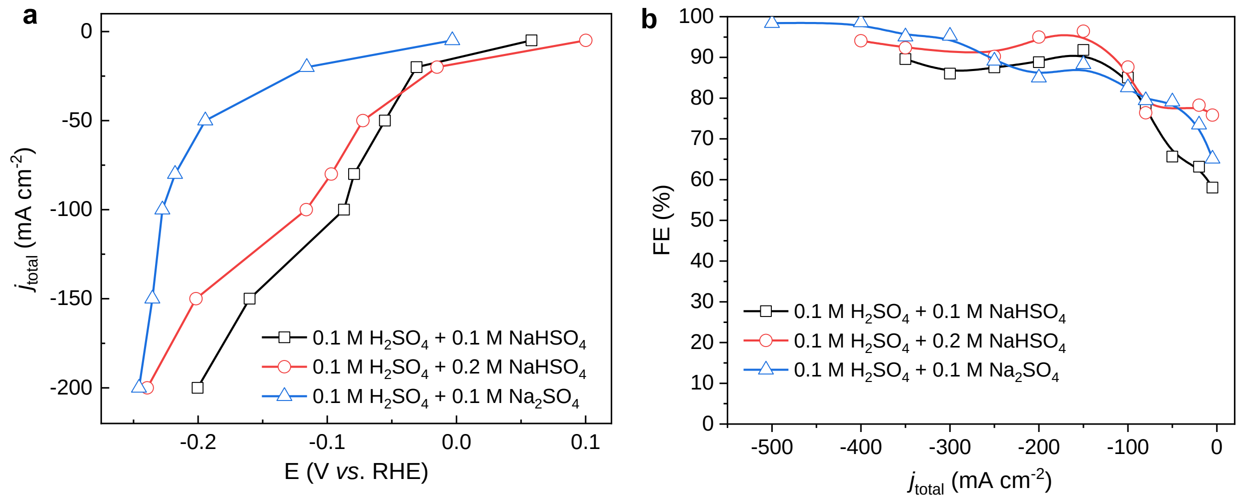

Figure S14. (**a**) The I-V curve and (**b**) FE of H_2O_2 production through 2e$^-$-ORR in 0.1 M H_2SO_4 by using $NaHSO_4$ and Na_2SO_4 as additives.

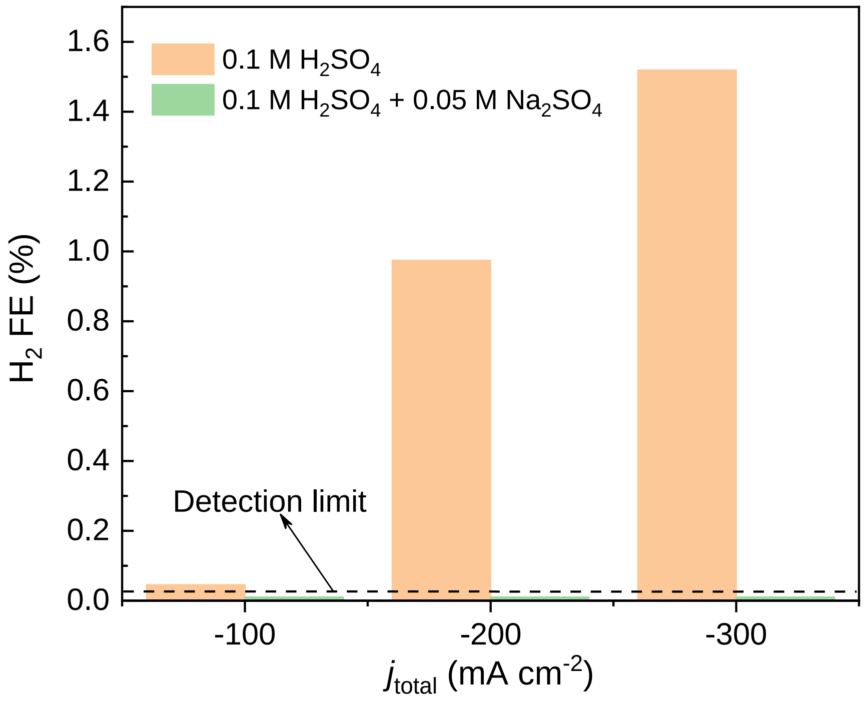

Figure S15. The FE of H_2 production during ORR with/without alkali metal cations (Na^+, Cs^+) as additives. The amount of H_2 produced was detected by GC. As shown in Figure S15, the H_2 amount are quite low, even at high current densities (low negative potential), indicating that the electrochemical process still proceeds through the ORR process. Therefore, in our theoretical simulation, we did not consider the HER process. In addition, it is clear that with the presence of cations (Na^+, Cs^+), the amount of H_2 produced is under the detection limit of the GC and the H_2 production through HER is inhibited.

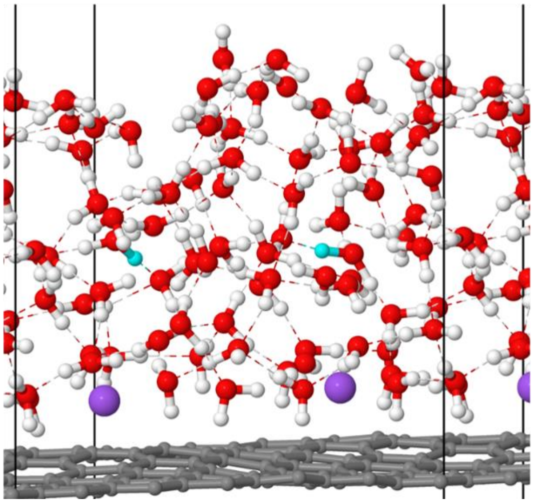

Figure S16. Representative periodic model for modelling cation/proton distribution under V_{RHE} = -1V. Na^+ is in purple, proton is in cyan, O atoms are in red, and all other hydrogen atoms are in white.

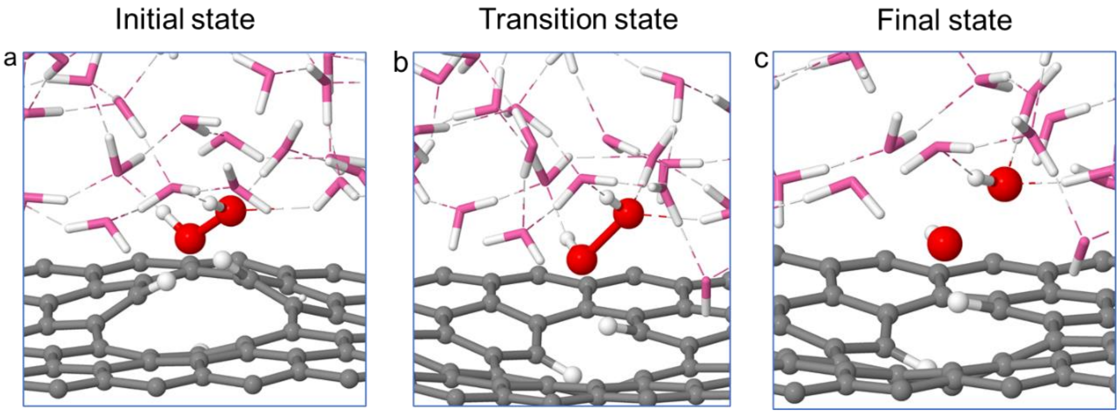

Figure S17. (a-c) Initial state, transition state and final state of the H_2O_2 decomposition in acid solution. C: grey; O: red; H: white; Proton: cyan.

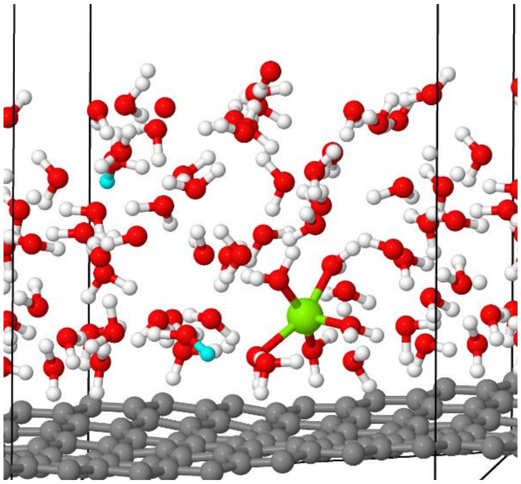

Figure S18. Final structure of the electrical double layer of water with Mg^{2+} and $2H^{+}$ under V_{RHE} = -1V after running AIMD for more than 4 picoseconds. The H is colored with cyan while the Mg is colored with green.

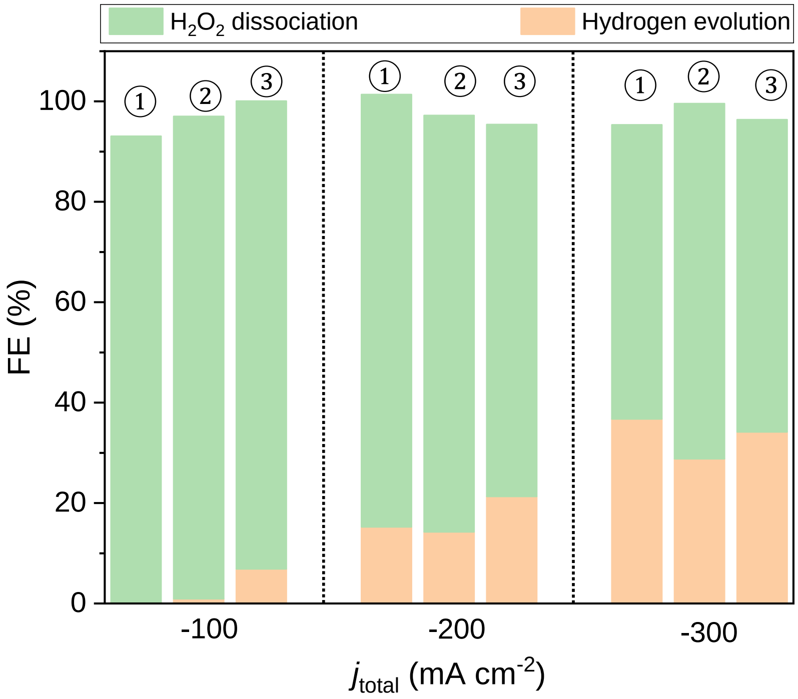

Figure S19. The FE of H_2O_2 dissociation in an H-cell at different current densities. The concentration of H_2O_2 is 0.2 M. The electrolyte is the mixture of 0.1 M H_2SO_4 + 0.2 M H_2O_2 solution. 0.05 M Na_2SO_4 or 0.05 M Cs_2SO_4 were incorporated into the electrolyte as alkali metal cation additives. 1 represents the reaction in the electrolyte without alkali metal cations, 2 represents the reaction in the electrolyte with Na^+ as additive, and 3 represents the reaction in the electrolyte with Cs^+ as additive.

During the H_2O_2 dissociation process, there are two electrochemical reactions at the cathode:

$$H_2O_2 + 2e^- + 2H^+ = 2H_2O \quad (1)$$

$$2H^+ + 2e^- = H_2 \quad (2)$$

It is clear from Fig. S19 that the total FE of the two reactions, i.e., H_2O_2 dissociation (equation 1) and hydrogen evolution (equation 2), is close to 100 %. Therefore, we can calculate the partial current density of H_2O_2 dissociation at the cathode through the exact measurement of H_2 formed during the H_2O_2 dissociation process.

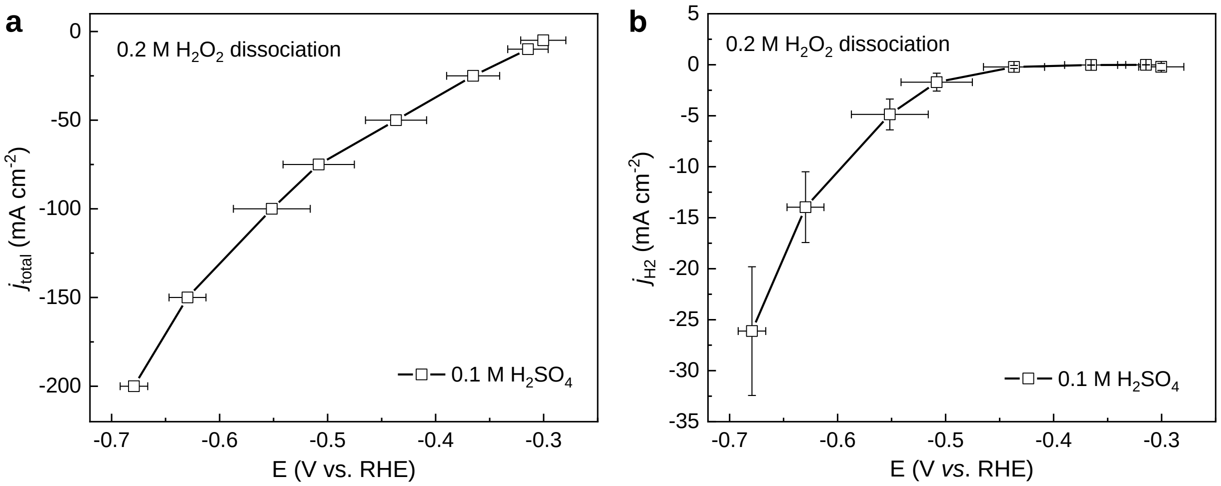

Figure S20. (**a**) The overall current density of H_2O_2 dissociation as a function of voltage in 0.1 M H_2SO_4 electrolyte. (**b**) The partial current density of H_2 production at the cathode as a function of voltage in 0.1 M H_2SO_4 electrolyte. The measured potentials were manually 100% compensated. The error bars represent two independent tests.

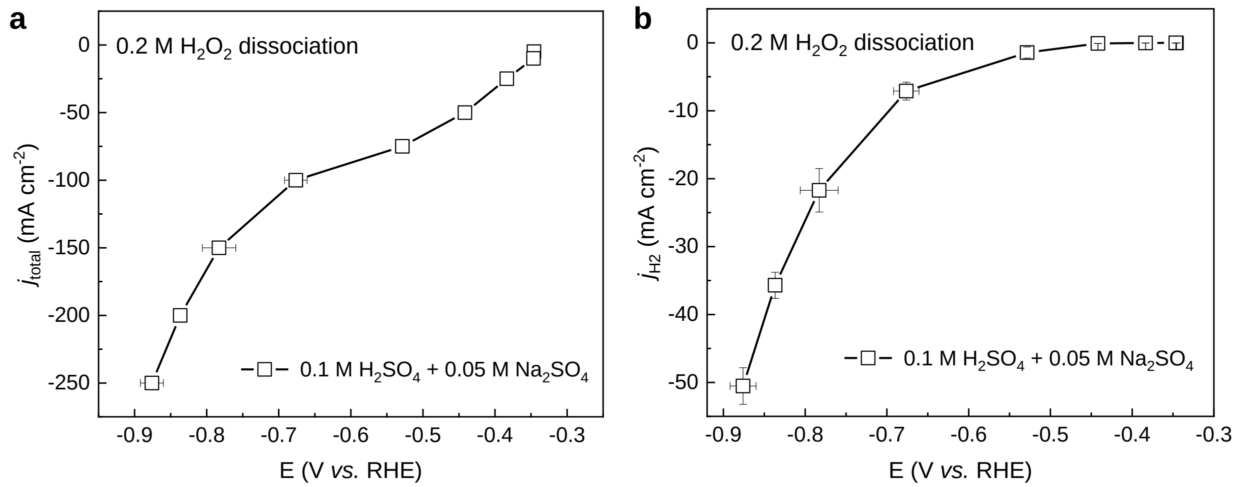

Figure S21. (**a**) The overall current density of H_2O_2 dissociation as a function of voltage in 0.1 M H_2SO_4 + 0.05 M Na_2SO_4 electrolyte. (**b**) The partial current density of H_2 production at the cathode as a function of voltage in 0.1 M H_2SO_4 + 0.05 M Na_2SO_4 electrolyte. The measured potentials were manually 100% compensated. The error bars represent two independent tests.

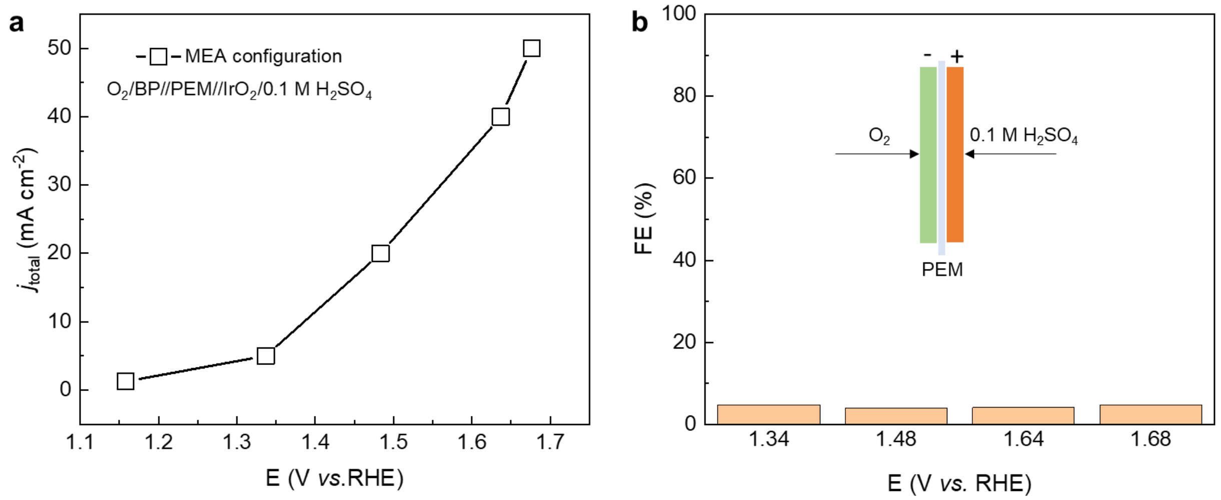

Figure S22. (**a**) The I-V curve and (**b**) FE of H_2O_2 production using a traditional membrane electrode assembly (MEA) cell with configuration of O_2+H_2O/BP2000//PEM//IrO_2/0.1 M H_2SO_4. The O_2 + H_2O mixture was supplied to the cathode and 0.1 M H_2SO_4 is used as the anolyte. The measured potentials were manually 100% compensated.

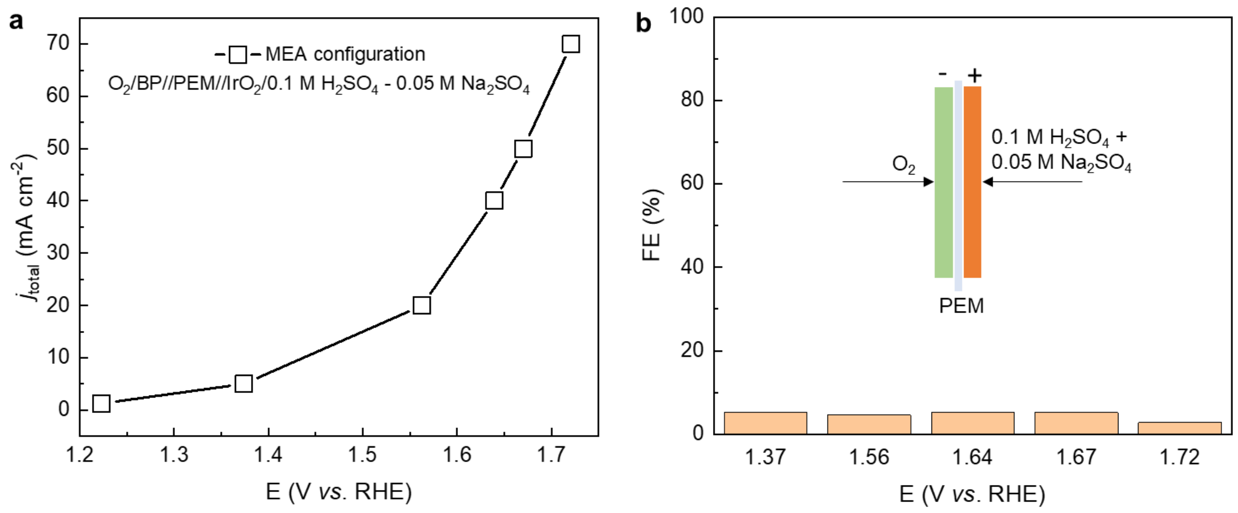

Figure S23. (**a**) The I-V curve and (**b**) FE of H_2O_2 production using MEA with configurations of O_2+H_2O/BP//PEM//IrO_2/0.1 M H_2SO_4-0.05 M Na_2SO_4. The O_2 + H_2O mixture was supplied to the cathode and the mixed solution of 0.1 M H_2SO_4 + 0.05 M Na_2SO_4 was used as the anolyte. The Na^+ ions in the anolyte are supposed to cross over the PEM and improve the H_2O_2 selectivity during the process. However, due to the high acidic property of Nafion-117, the local proton concentration is still high and the proton dominates the ion transportation process (Fig. S23, S24). The H_2O_2 selectivity remains at a low level at all potentials. The measured potentials were manually 100% compensated.

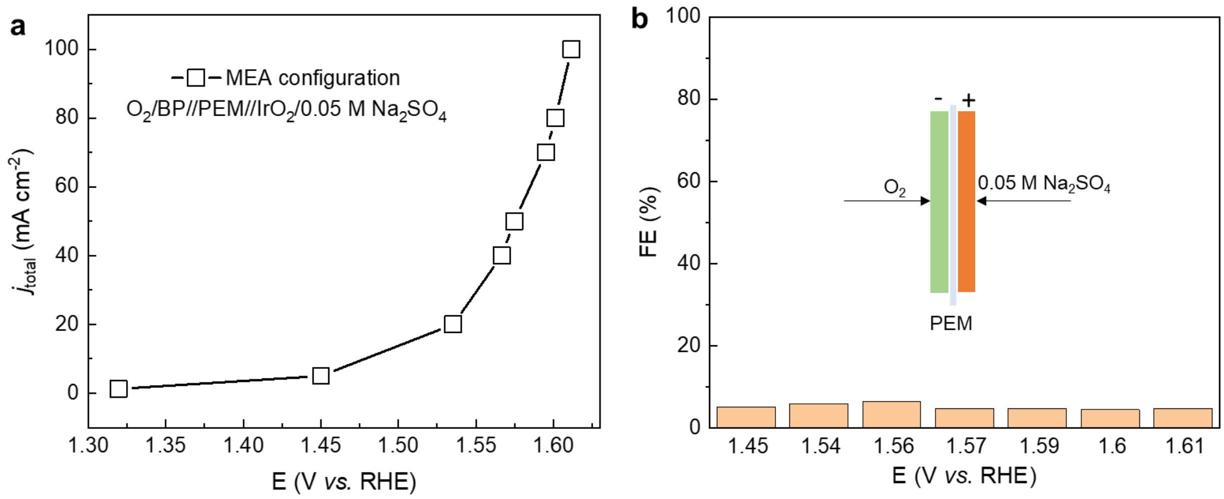

Figure S24. (**a**) The I-V curve and (**b**) FE of H_2O_2 production using MEA with configurations of O_2+H_2O/BP//PEM//IrO_2/0.05M Na_2SO_4. The O_2+H_2O mixture was supplied to the cathode, and the solution of 0.05 M Na_2SO_4 is used as the anolyte. The measured potentials were manually 100% compensated.

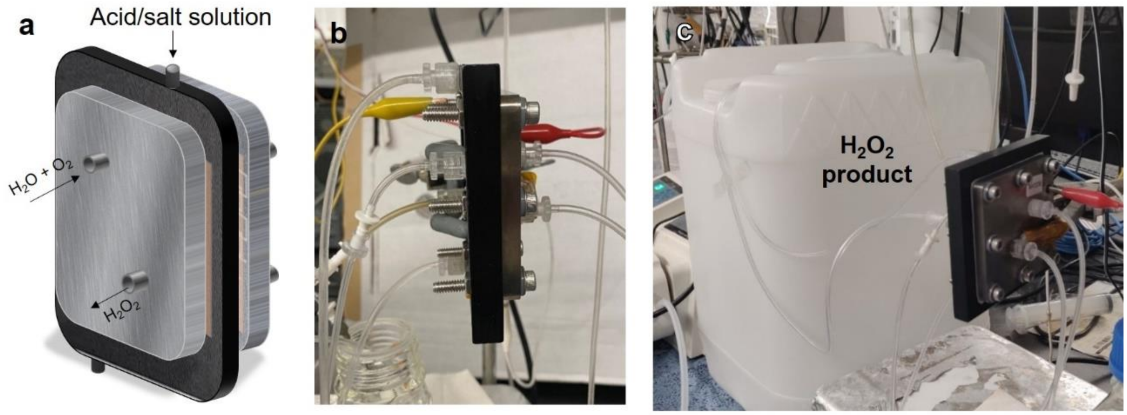

Figure S25: (**a**) The schematic illustration of the SE electrochemical cell with double-PEM configuration. (**b, c**) Photographs of the setup for producing H_2O_2 using the SE cell.

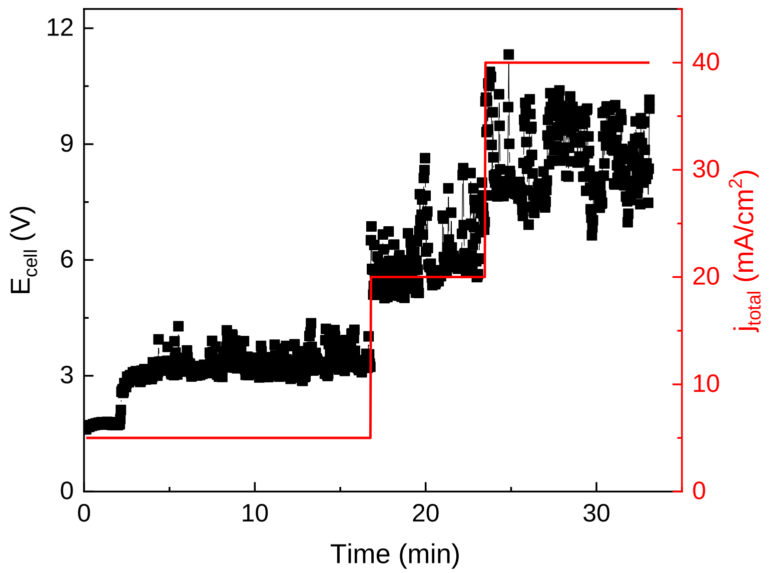

Figure S26. The I-V curve of the cell without SE in the middle channel. 0.03 M Na_2SO_4 flows into the middle channel. Without the ion-conductive SE, the catalytic process has high resistance, giving high and unstable voltages during the constant current electrolysis.

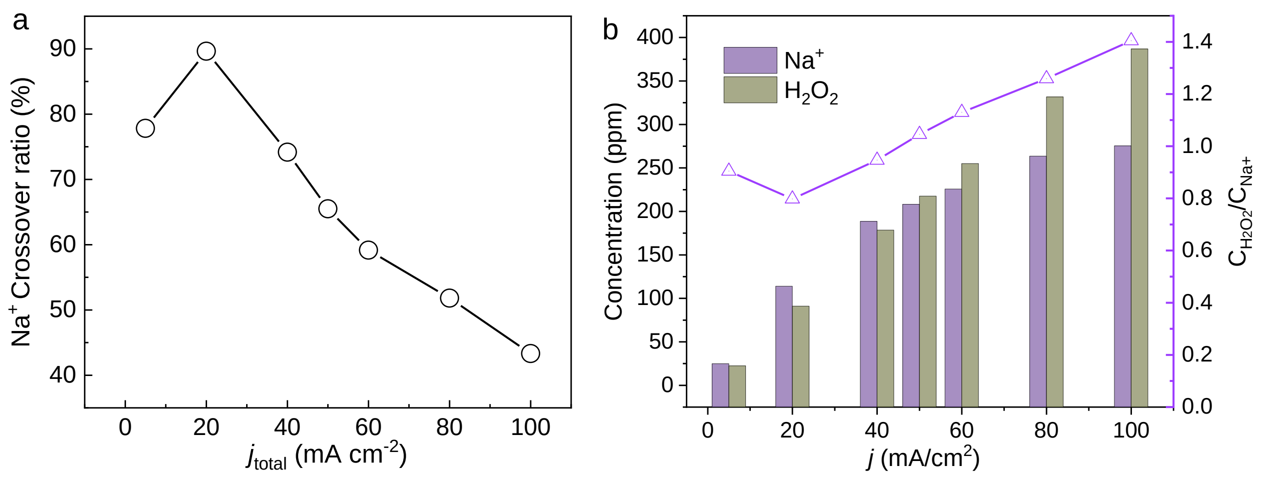

Figure S27. (a) The Na^{+} cross-over ratio through the cathodic PEM in the SE cell by using 0.03 M Na_2SO_4 in the middle chamber. **(b)** The correlation of the crossover of Na^{+} with the production rate of H_2O_2. The concentration and concentration ratio in Fig. S27b are based on the mass concentration of H_2O_2 and Na^{+} tested from ICP.

Supplementary Note 2:

Similar to flow cell, the alkali metal cation in the middle chamber is important for selectively producing H_2O_2 at high current density. At a negative potential, the cations in the middle chamber penetrate the PEM closest to the cathode side and move to the catalyst surface in the SE cell (the absence of PEM at the cathode side will induce strong flooding during operation). To quantify the cross over Na^+ toward H_2O_2 production in our SE cell, the concentration of H_2O_2 product and crossover Na^+ from cathode side were analyzed as a function of the applied current. As shown in Fig. S27, the Na^+ crossover ratio is first increased at 20 mA cm^{-2} and then decreased along with increasing current density, reaching 43% at 100 mA cm^{-2}. The trend is reversed with the concentration ratio of H_2O_2 to Na^+. Moreover, the Na^+ crossover ratio is closely related to the current densities applied in the cell. At a small current, the Na^+ penetrated is enough to maintain the local catalytic environment with "neutral", thus guaranteeing a high H_2O_2 selectivity. However, upon increasing the current density, the Na^+ crossover ratio decreases and protons might participate in the process. We suppose that once the value decreases to ~ 50 %, the H_2O_2 FE starts to decrease because the low concentration of Na^+ is not enough to maintain the local environment for H_2O_2 production and protons start to dominate near the electrode.

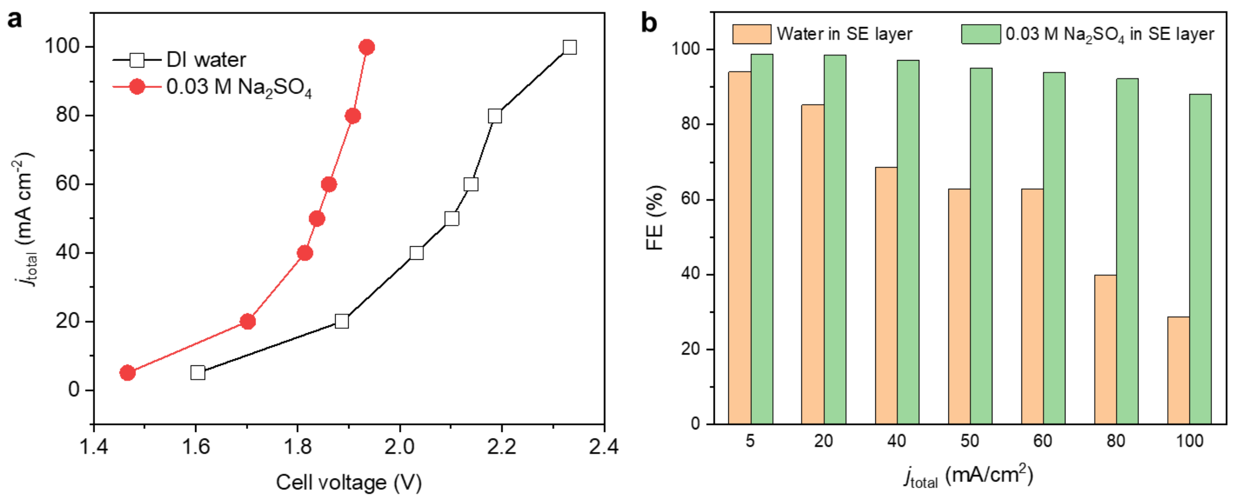

Figure S28. (**a**) The I-V curve of ORR in the SE cell with the double-PEM configuration by flowing DI water and 0.03 M Na_2SO_4 in the middle chamber. (**b**) The corresponding FE of production of H_2O_2 as a function of current densities. The presence of Na^+ cations in the middle chamber improves the activity and selectivity of H_2O_2 during the production process in the SE cell with a double-PEM configuration. The measured potentials were manually 100% compensated.

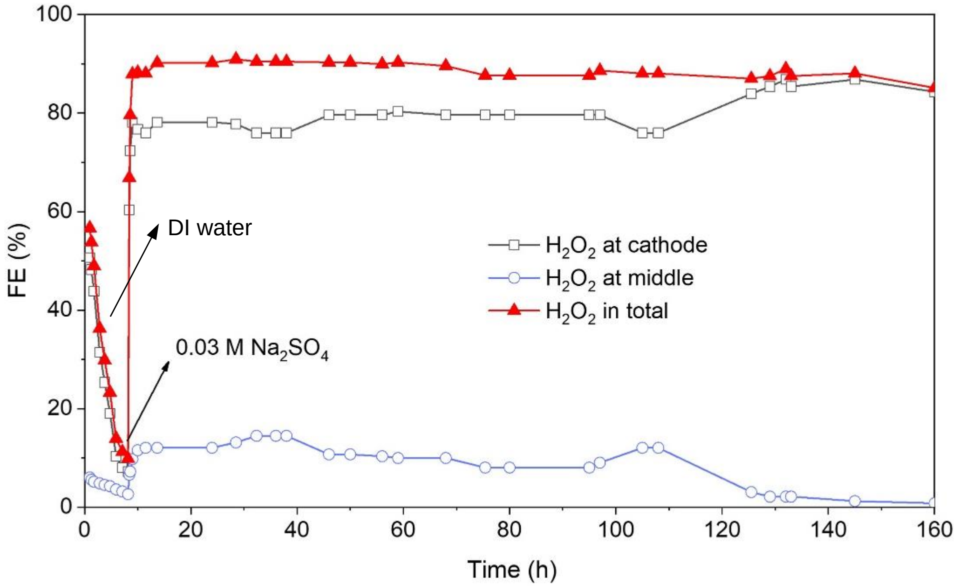

Figure S29. The effect of alkali metal cations toward the H_2O_2 production stability in the double-PEM SE cell. Without cations, the FE of H_2O_2 continuously declines to less than 10% within 6 h, while the addition of 0.03 M Na_2SO_4 in the middle chamber improves the FE of H_2O_2. The FE of H_2O_2 production with 0.03 M Na_2SO_4 in the middle chamber is well maintained, suggesting a continuous and stable generation of H_2O_2 solutions.

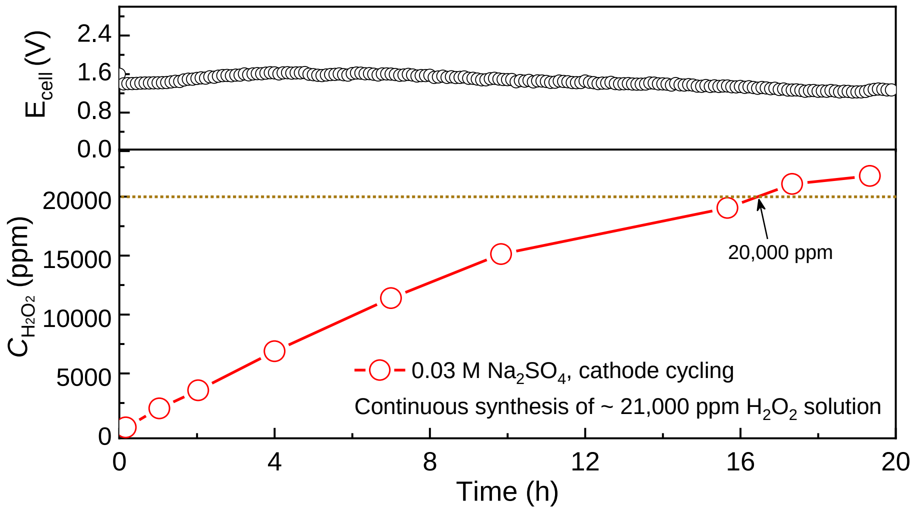

Figure S30. Time-dependent H_2O_2 concentration measured at a constant current density of 50 mA cm^{-2} (total current 200 mA) with 0.03 M Na_2SO_4 as electrolyte in the middle chamber of the SE cell. The accumulated H_2O_2 concentration reaches a metric value of ~20,000 ppm after 17 h of electrolysis in 50 mL when the product is continuously cycled through the system. The measured potentials were manually 100% compensated.

Supplementary Table 1. Selected set-up for cathodic electrosynthesis of H_2O_2 from oxygen. The current densities are collected by eyeballing from the current-potential (I-V) curves. The current densities in RRDE represent the disk current densities.

Electrocatalyst	Electrolytes	Testing Tools	Efficiency (H_2O_2 %)@mA/cm^2	Ref
N-doped Carbon Nanohorns	0.1M H_2SO_4	RRDE	~98 @ 0.7	7
F-doped Porous Carbon	0.05 M H_2SO_4	RRDE	~97.5–83.0 @ 1-2.5^{R}	8
N-doped Mesoporous Carbon	0.5 M H_2SO_4	RRDE	~95–98% @ 0.3-1^{R}	9
Single atomic Pt-CuS_x	0.1 M $HClO_4$	RRDE	~92-96 @ 0.1-2.8^{R}	10
Pt_1/TiN	0.1 M $HClO_4$	RRDE	~90 @ <0.05^{R}	11
Co-N-C	0.1 M $HClO_4$	RRDE	>90% @ 1^{R}	12
CoS_2	0.05 M H_2SO_4	RRDE	~80% @ 0.7	13
	0.05 M H_2SO_4	H-cell	70.6 @ 1.7	
$PtHg_4$/C	0.1 M $HClO_4$	RRDE	96 @ 3	14
Pd_2Hg_5/C	0.1 M $HClO_4$	RRDE	95 @ 0.7	15
$Au_{92}Pd_8$/C	0.1 M $HClO_4$	RRDE	95@ 1.5	16
Carbon-coated Pt	1 M $HClO_4$	RRDE	41@1.8	17
Atomically Pt on carbon	1 M $HClO_4$	RRDE	96 @ 1.5	18
Hierarchically Porous Carbon	H_2SO_4+ Na_2SO_4 (pH = 1)	RRDE	95 @ 0 .8	19
	H_2SO_4+ Na_2SO_4 (pH = 1)	Two compartment H-cell	85.2-91.2 @ -0.1~-0.3V	
PdP_2	0.1 M $HClO_4$	RRDE	~98.5 @ 3^{R}	20
	Water/attached with Nafion	PEMFC	~78.8 @ 150	

References

(1) Zhu, S.; Hu, X.; Zhang, L.; Shao, M. Impacts of Perchloric Acid, Nafion, and Alkali Metal Ions on Oxygen Reduction Reaction Kinetics in Acidic and Alkaline Solutions. *J. Phys. Chem. C* **2016,** *120* (48), 27452.

(2) Kumeda, T.; Tajiri, H.; Sakata, O.; Hoshi, N.; Nakamura, M. Effect of hydrophobic cations on the oxygen reduction reaction on single–crystal platinum electrodes. *Nat. Commun.* **2018,** *9* (1), 4378.

(3) Strmcnik, D.; Kodama, K.; van der Vliet, D.; Greeley, J.; Stamenkovic, V. R.; Marković, N. M. The role of non-covalent interactions in electrocatalytic fuel-cell reactions on platinum. *Nat. Chem.* **2009,** *1* (6), 466.

(4) Okada, T.; Ayato, Y.; Satou, H.; Yuasa, M.; Sekine, I. The Effect of Impurity Cations on the Oxygen Reduction Kinetics at Platinum Electrodes Covered with Perfluorinated Ionomer. *J. Phys. Chem. B* **2001,** *105* (29), 6980.

(5) Sun, Y.; Silvioli, L.; Sahraie, N. R.; Ju, W.; Li, J.; Zitolo, A.; Li, S.; Bagger, A.; Arnarson, L.; Wang, X.et al. Activity–Selectivity Trends in the Electrochemical Production of Hydrogen Peroxide over Single-Site Metal–Nitrogen–Carbon Catalysts. *J. Am. Chem. Soc.* **2019,** *141* (31), 12372.

(6) Yang, S.; Verdaguer-Casadevall, A.; Arnarson, L.; Silvioli, L.; Čolić, V.; Frydendal, R.; Rossmeisl, J.; Chorkendorff, I.; Stephens, I. E. L. Toward the Decentralized Electrochemical Production of H_2O_2: A Focus on the Catalysis. *ACS Catal.* **2018,** *8* (5), 4064.

(7) Iglesias, D.; Giuliani, A.; Melchionna, M.; Marchesan, S.; Criado, A.; Nasi, L.; Bevilacqua, M.; Tavagnacco, C.; Vizza, F.; Prato, M.et al. N-Doped Graphitized Carbon Nanohorns as a Forefront Electrocatalyst in Highly Selective O_2 Reduction to H_2O_2. *Chem* **2018,** *4* (1), 106.

(8) Zhao, K.; Su, Y.; Quan, X.; Liu, Y.; Chen, S.; Yu, H. Enhanced H_2O_2 production by selective electrochemical reduction of O_2 on fluorine-doped hierarchically porous carbon. *J. Catal.* **2018,** *357*, 118.

(9) Sun, Y.; Sinev, I.; Ju, W.; Bergmann, A.; Dresp, S.; Kühl, S.; Spöri, C.; Schmies, H.; Wang, H.; Bernsmeier, D.et al. Efficient Electrochemical Hydrogen Peroxide Production from

Molecular Oxygen on Nitrogen-Doped Mesoporous Carbon Catalysts. *ACS Catal.* **2018,** *8* (4), 2844.

(10) Shen, R.; Chen, W.; Peng, Q.; Lu, S.; Zheng, L.; Cao, X.; Wang, Y.; Zhu, W.; Zhang, J.; Zhuang, Z.et al. High-Concentration Single Atomic Pt Sites on Hollow CuS_x for Selective O_2 Reduction to H_2O_2 in Acid Solution. *Chem* **2019,** *5* (8), 2099.

(11) Yang, S.; Kim, J.; Tak, Y. J.; Soon, A.; Lee, H. Single-Atom Catalyst of Platinum Supported on Titanium Nitride for Selective Electrochemical Reactions. *Angew. Chem. Int. Ed.* **2016,** *55* (6), 2058.

(12) Gao, J.; Yang, H. b.; Huang, X.; Hung, S.-F.; Cai, W.; Jia, C.; Miao, S.; Chen, H. M.; Yang, X.; Huang, Y.et al. Enabling Direct H_2O_2 Production in Acidic Media through Rational Design of Transition Metal Single Atom Catalyst. *Chem* **2020,** *6* (3), 658.

(13) Sheng, H.; Hermes, E. D.; Yang, X.; Ying, D.; Janes, A. N.; Li, W.; Schmidt, J. R.; Jin, S. Electrocatalytic Production of H_2O_2 by Selective Oxygen Reduction Using Earth-Abundant Cobalt Pyrite (CoS_2). *ACS Catal.* **2019,** *9* (9), 8433.

(14) Siahrostami, S.; Verdaguer-Casadevall, A.; Karamad, M.; Deiana, D.; Malacrida, P.; Wickman, B.; Escudero-Escribano, M.; Paoli, E. A.; Frydendal, R.; Hansen, T. W.et al. Enabling direct H_2O_2 production through rational electrocatalyst design. *Nat. Mater.* **2013,** *12* (12), 1137.

(15) Verdaguer-Casadevall, A.; Deiana, D.; Karamad, M.; Siahrostami, S.; Malacrida, P.; Hansen, T. W.; Rossmeisl, J.; Chorkendorff, I.; Stephens, I. E. L. Trends in the Electrochemical Synthesis of H_2O_2: Enhancing Activity and Selectivity by Electrocatalytic Site Engineering. *Nano Lett.* **2014,** *14* (3), 1603.

(16) Jirkovský, J. S.; Panas, I.; Ahlberg, E.; Halasa, M.; Romani, S.; Schiffrin, D. J. Single Atom Hot-Spots at Au–Pd Nanoalloys for Electrocatalytic H_2O_2 Production. *J. Am. Chem. Soc.* **2011,** *133* (48), 19432.

(17) Choi, C. H.; Kwon, H. C.; Yook, S.; Shin, H.; Kim, H.; Choi, M. Hydrogen Peroxide Synthesis via Enhanced Two-Electron Oxygen Reduction Pathway on Carbon-Coated Pt Surface. *J. Phys. Chem. C* **2014,** *118* (51), 30063.

(18) Choi, C. H.; Kim, M.; Kwon, H. C.; Cho, S. J.; Yun, S.; Kim, H.-T.; Mayrhofer, K. J. J.; Kim, H.; Choi, M. Tuning selectivity of electrochemical reactions by atomically dispersed platinum catalyst. *Nat. Commun.* **2016,** *7* (1), 10922.

(19) Liu, Y.; Quan, X.; Fan, X.; Wang, H.; Chen, S. High-Yield Electrosynthesis of Hydrogen Peroxide from Oxygen Reduction by Hierarchically Porous Carbon. *Angew. Chem. Int. Ed.* **2015,** *54* (23), 6837.

(20) Li, H.; Wen, P.; Itanze, D. S.; Hood, Z. D.; Adhikari, S.; Lu, C.; Ma, X.; Dun, C.; Jiang, L.; Carroll, D. L.et al. Scalable neutral H_2O_2 electrosynthesis by platinum diphosphide nanocrystals by regulating oxygen reduction reaction pathways. *Nat. Commun.* **2020,** *11* (1), 3928.
